# A systematic review of fMRI neurofeedback reporting and effects in clinical populations

**DOI:** 10.1016/j.nicl.2020.102496

**Published:** 2020-11-11

**Authors:** Anita Tursic, Judith Eck, Michael Lührs, David E.J. Linden, Rainer Goebel

**Affiliations:** a Department of Cognitive Neuroscience, Faculty of Psychology and Neuroscience, Maastricht University, Oxfordlaan 55, 6229 EV Maastricht, The Netherlands; b Brain Innovation B.V, Oxfordlaan 55, 6229 EV Maastricht, The Netherlands; c School for Mental Health and Neuroscience, Faculty of Health, Medicine and Life Sciences, Maastricht University, Universiteitssingel 40, 6229 ER Maastricht, The Netherlands; d Department of Neuroimaging and Neuromodeling, Netherlands Institute for Neuroscience, Meibergdreef 47, 1105 BA Amsterdam, the Netherlands

**Keywords:** Real-time fMRI, Self-regulation, Power, Effect size, Mental disorders, Clinical trial

## Abstract

Real-time fMRI-based neurofeedback is a relatively young field with a potential to impact the currently available treatments of various disorders. In order to evaluate the evidence of clinical benefits and investigate how consistently studies report their methods and results, an exhaustive search of fMRI neurofeedback studies in clinical populations was performed. Reporting was evaluated using a limited number of Consensus on the reporting and experimental design of clinical and cognitive-behavioral neurofeedback studies (CRED-NF checklist) items, which was, together with a statistical power and sensitivity calculation, used to also evaluate the existing evidence of the neurofeedback benefits on clinical measures. The 62 found studies investigated regulation abilities and/or clinical benefits in a wide range of disorders, but with small sample sizes and were therefore unable to detect small effects. Most points from the CRED-NF checklist were adequately reported by the majority of the studies, but some improvements are suggested for the reporting of group comparisons and relations between regulation success and clinical benefits. To establish fMRI neurofeedback as a clinical tool, more emphasis should be placed in the future on using larger sample sizes determined through *a priori* power calculations and standardization of procedures and reporting.

## Highlights

• Standardization of fMRI neurofeedback methods and results reporting is imperative.
• Currently available reports of crucial measures are limited.
• Past clinical fMRI neurofeedback studies could detect medium and big effect sizes.
• Studies are limited by small sample sizes in different disorders.

## 1 Introduction

Neurofeedback uses measured changes in brain activation to help participants regulate the activity (in selected regions or networks) or power of selected EEG frequency bands by providing them with the activation information in real time. Neurofeedback can be conducted with a variety of neuroimaging techniques, such as fMRI (Thibault et al., 2018), EEG (Micoulaud-Franchi et al., 2015), and fNIRS (Kohl et al., 2020), each selected based on the combination of the research question and the technique’s specific advantages; this includes, but is not limited to spatial coverage, spatial and temporal resolution, portability, costs, and general ease of use (Liu et al., 2016, Thibault et al., 2015). The present review is focusing on fMRI, which offers superior spatial resolution and whole-brain coverage but has the disadvantage of availability, cost, and non-portability, i.e. it cannot be performed at home or at a patient’s bedside.

fMRI neurofeedback is a relatively novel method, dating back to 2003, when the first exemplary data was published (Weiskopf et al., 2003). It has become possible with the development of real-time analysis options and has quickly gained interest because of its high spatial resolution and whole-brain coverage (Sulzer et al., 2013, Thibault et al., 2018, Watanabe et al., 2017, Weiskopf et al., 2004). First, the region of interest (or connectivity between regions (Liew et al., 2016, Pereira et al., 2019, Ramot et al., 2017)) is determined based on the behavioral changes that are expected to result from neurofeedback training. This can be done with a functional localizer using a task that closely resembles the targeted behavior or using anatomical information based on previous research. The neurofeedback part then guides the participants to improve their region-of-interest activation (or network connectivity) control by informing them about their performance in real time. The activation-based feedback usually represents a signal change between periods of self-regulation and the preceding rest period, whereas connectivity-based neurofeedback shows the changes in the correlation between regions or their coupling, for example on the basis of correlation coefficients or parameter estimates from dynamic causal modeling (DCM) (Watanabe et al., 2017).

Furthermore, although the studies presented in the present review focus on univariate analysis of region-of-interest activation or connectivity regulation described above, it is possible to also use decoded neurofeedback (DecNef), which uses a multivariate approach (i.e., spatial patterns of activity (LaConte, 2011, LaConte et al., 2007)). The provided feedback therefore does not present the achieved activation or connectivity change, but the likelihood that the participant has achieved the predetermined target brain activity pattern. The achieved activation pattern in each trial is compared to the target, predetermined pattern; then the participant is notified how similar their pattern is to the target one, but without any explicit knowledge of the task. The main goal of the training is therefore to learn how to elicit this predetermined state (Shibata et al., 2019, Watanabe et al., 2017).

### 1.1 Applications of neurofeedback

Neurofeedback is used in healthy participants and clinical populations for cognitive performance enhancement training or as a clinical intervention, respectively. The targeted behavioral changes vary from improved working memory (Sherwood et al., 2016, Zhang et al., 2015), increased motor performance (Hui et al., 2014, Scharnowski et al., 2015) and decreased pain perception (Rance et al., 2014), to decreased clinical symptoms, for example in depression (Linden et al., 2012, Mehler et al., 2018, Young et al., 2017b, Young et al., 2017a) or PTSD (Gerin et al., 2016, Zotev et al., 2018).

Over the last 10 years, research into clinical applications of fMRI neurofeedback in psychiatry and neurorehabilitation has expanded considerably, among other reasons, because of the increasing disease burden in these fields of medicine and the difficulties in treating many of the often-chronic conditions they cover. Neurofeedback, if proven sufficiently efficacious and effective (i.e., in ideal, controlled clinical environment, and practically, in “real world”, respectively (Godwin et al., 2003)) considering its cost/benefit ratio, could become an alternative or add-on treatment in the future.

Dropouts, treatment resistance, and side effects are relevant examples of why exploring new methods that might relieve symptoms in clinical populations is important. For example, in post-traumatic stress disorder (PTSD), the dropout for two standard treatments, namely cognitive behavioral therapy (CBT) and eye movement desensitization and reprocessing (EMDR), ranges between 19 and 27% (Hembree et al., 2003) or even up to 38% (Schnurr et al., 2007).

Treatment resistance and side effects are factors limiting many pharmacological treatments in psychiatry. For example, antidepressants show effects when first prescribed in only about 30% of the patients, with the majority of patients needing a change or added medication or cognitive behavioral therapy multiple times to achieve remission (Gaynes et al., 2009, Warden et al., 2007). In addition, antidepressants are associated with several adverse effects (Kennedy et al., 2016, Qaseem et al., 2016), making alternative treatment options desirable.

### 1.2 Quality measures of neurofeedback research

The effectiveness and clinical potential of neurofeedback can be efficiently evaluated when sufficient and unified methods are used, and results reported. Currently available guidelines for rt-fMRI-NF studies, such as CRED-NF (Ros et al., 2020) and TIDieR (Randell et al., 2018), recommend pre-registering studies, standardized and robust measures, designs and statistical analysis, and clear reporting.

A good study design, solid methodology and transparent reporting of results are crucial for evaluating whether fMRI neurofeedback can become a supportive or even stand-alone treatment for various disorders. A large number of reviews has already investigated effects of fMRI neurofeedback, but they mainly focused on a specific disorder (Chiba et al., 2019, Frank et al., 2013, Gonçalves et al., 2017, Linden and Turner, 2016) or neurofeedback effects in general (Brühl, 2015, deCharms, 2007, Scharnowski and Weiskopf, 2015, Weiskopf, 2012). Recently, a critical systematic review of all rt-fMRI-NF studies has been published (Thibault et al., 2018), but an exhaustive systematic review of clinical effects of neurofeedback is still missing. Therefore, the present systematic review focuses solely on fMRI neurofeedback performed on clinical populations and investigates the consistency of reporting with regards to the current guidelines and clinical benefits. In particular, published studies are evaluated based on whether the CRED-NF guidelines are applied, design similarities are investigated, and the consistency of results is reported. We do recognize that the CRED-NF checklist has only been published recently, so the vast majority, if not all publications included in the present review, could not have used it for their reporting; the checklist is therefore merely used to evaluate the reporting practices of extant fMRI neurofeedback studies. Furthermore, in order to evaluate the level of evidence for the effects of neurofeedback and the progress of research, an estimation of the overall statistical power and sensitivity for small, medium, and large effect sizes are calculated.

## 2 Methods

### 2.1 Publication search

We included several online sources of data to ensure completeness: PubMed (www.ncbi.nlm.nih.gov/pubmed), Web of Science (www.webofknowledge.com), Arxiv (https://arxiv.org), BioRxiv (https://www.biorxiv.org), MedRxiv (https://www.medrxiv.org), PsyArXiv (https://psyarxiv.com), Open Science Framework (https://osf.io). Two different international databases of registered clinical trials were also included in the search of literature and clinical trials: ISCTN (https://www.isrctn.com) and ClinicalTrials.gov (https://clinicaltrials.gov).

Finally, references from identified original clinical research papers and review articles were checked and remaining relevant scientific publications were added.

**Fig. 1:**
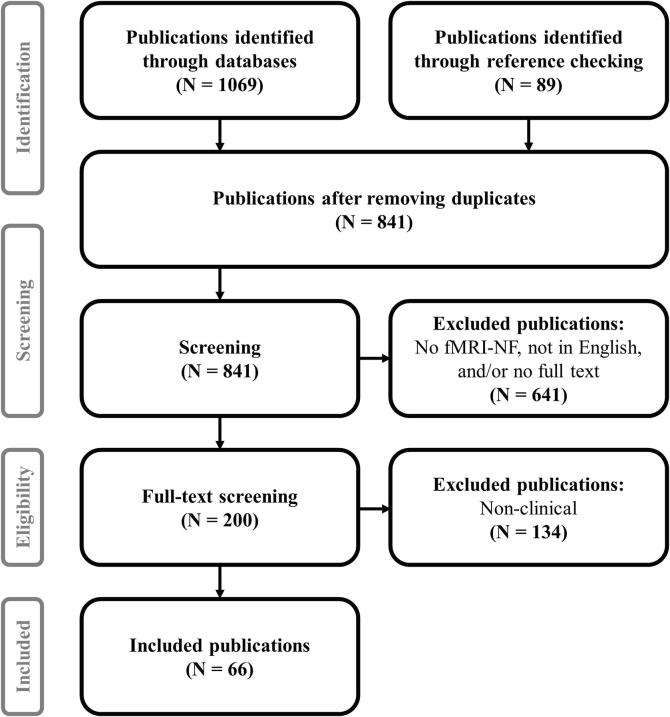
Search protocol. The search for publications was conducted following PRISMA guidelines (Moher et al., 2009).

The search was conducted in English and followed the PRISMA guidelines (Moher et al., 2009), see Fig. 1. The keywords used were “neurofeedback AND fMRI”, meaning we were looking for publications that mentioned both neurofeedback and fMRI.

General exclusion criteria for the literature search were publications in a language other than English and publications published before the year 2000, considering that the first exemplary data was published in 2003 (Weiskopf et al., 2003). References without an available full text (abstracts and conference/meeting abstracts) were excluded. Studies that did not report fMRI neurofeedback results (e.g., other modalities, such as EEG, studies not conducted in real-time, or methods papers) were excluded.

Publications were excluded following the analysis of the abstract if the abstract showed no relevance for the current review. If the abstract showed potential relevance, the full text of the publication was carefully read, and its relevance was either confirmed or the publication was excluded.

Out of 1158 hits, 317 duplicates (i.e., found in more than one search engine) were removed and the exclusion criteria were applied. 200 publications were left, including both healthy and clinical populations.

Finally, publications with only healthy participants were excluded. 66 publications were left, including only fMRI neurofeedback studies in a clinical population.

### 2.2 General approach

To provide a general overview of the publication trends, the studies were first sorted based on the publication year.

Due to the high variability of investigated disorders, publications with clinical populations were then sorted into different groups based on ICD-10 (The ICD-10 classification of mental and behavioral disorders: diagnostic criteria for research, 1993) and DSM-5 (Diagnostic and statistical manual of mental disorders: DSM-5, 5th ed., 2013) criteria. Based on this, Alzheimer’s, Huntington’s, and Parkinson’s disease were grouped into a neurodegenerative disorders group. Hemineglect, hemiparalysis and stroke were included in the brain damage group. Contamination anxiety (as a trait of obsessive–compulsive disorder (OCD)) and arachnophobia were grouped into an anxiety disorder group. The addiction group included different substances, namely alcohol, nicotine, and cocaine. Finally, some publications investigated various disorders in the same analysis. These were grouped into miscellaneous disorders. The rest of the disorders were kept in their own groups: attention deficit hyperactivity disorder (ADHD), autistic disorder, borderline personality disorder, chronic pain, depression, expressive aphasia, obesity, psychopathy, post-traumatic stress disorder (PTSD), schizophrenia, and tinnitus.

#### 2.2.1 CRED-NF checklist

To investigate the level of standardization of study design and reporting in the published studies, we followed the best practice recommendations set by the Consensus on the reporting and experimental design of clinical and cognitive-behavioral neurofeedback studies (CRED-NF checklist) (Ros et al., 2020). The checklist is aimed to be included with each neurofeedback study across different neurofeedback modalities and is also recommended for studies submitted to the present special issue.

We focused only on the *essential* checklist items and left out all the *encouraged* items, except for the preregistration (1a). We also left out the technical specifications (the Feedback specifications (4a-e) and point 3d regarding online preprocessing and artifact corrections), as they do not directly showcase the benefits of neurofeedback in clinical populations and are therefore not a part of this review’s main question. The final checklist therefore included 10 items that are listed in Table 1 and include pre-registration and sample size calculations, control groups and measures, such as blinding and strategies, and regulation and behavioral results reporting. Each study was summarized in Table 2 following the checklist items. Finally, the overall reporting of each checklist item was evaluated for all studies combined. The investigation of certain items was further extended once the preliminary results were collected; additional analysis steps and results are therefore described under each item in the results section, if applicable.

**Table 1:** Selected CRED-NF questions. The checklist items relevant for this review represent 10 out of 23 stated in the original CRED-NF checklist. Non-essential (encouraged) items and essential items related to technical details, such as data processing and feedback specifications, are omitted.

Domain	Item	Checklist item
Pre-experiment		
	1a	Pre-register experimental protocol and planned analyses
	1b	Justify sample size
Control groups		
	2a	Employ control group(s) or control condition(s)
	2b	When leveraging experimental designs where a double-blind is possible, use a double-blind
Control measures		
	3b	Report whether participants were provided with a strategy
Outcome measures		
Brain	5a	Report neurofeedback regulation success based on the feedback signal
	5b	Plot within-session and between-session regulation blocks of feedback variable(s), as well as pre-to-post resting baselines or contrasts
	5c	Statistically compare the experimental condition/group to the control condition(s)/group(s) (not only each group to the baseline measures)
Behavior	6a	Include measures of clinical or behavioral significance, defined a priori, and describe whether they were reached
	6b	Run correlational analyses between regulation success and behavioral outcomes

**Table 2:** Details of all 62 clinical fMRI neurofeedback studies found in an extensive search. Studies are sorted based on the clinical population and are categorized based on their methods and results. Next to each category, a corresponding item number from CRED-NF (see Table 1) is noted in brackets.

Publication (1b)	Clinical population	Clinical group size^3^	ROI	Strategy (3b)	Transfer run(s) and success	Control/comparison^11^ group (2a, 2b)	Regulation direction and success (5a)	Experimental vs control regulation (5c)	Regulation plots per run/session (5b)	Symptom measures (6a)	Regulation and clinical correlation (6b)	Follow-up	Registered trial (1a)
Canterberry et al., 2013	Addiction	9	ACC	No	No	–	↓ Yes	–	Sess	Beh ~	No	–	–
Hanlon et al., 2013	Addiction	15	vACC and dmPFC	No	No	–	↓ Yes	–	–	Beh –	–	–	–
Li et al., 2013 $	Addiction	12	ACC and mPFC	No	No	–	↓↑ _diff_ Yes (ACC)	–	–	Beh ↑	Yes	–	–
Karch et al., 2015 $	Addiction	13	ACC, dlPFC, or insula	No	No	Multi. Healthy NFB & SHAM, Clin SHAM ✗	↓ Yes	No, two groups	–	Clin ✗	–	–	–
Karch et al., 2019	Addiction	22^4^	ACC, dlPFC, or insula	No	No	Clin Diff ROI –	n.a.^13^	n.a.	–	Clin ✗	–	–	–
Kim et al., 2015	Addiction	7	# Bilateral ACC, medial pFC and OFC	No	Yes ✓	Clin Diff con ✓	↑ Yes	No (con > exp)	Sess	Beh ✗	–	–	–
Hartwell et al., 2016	Addiction	21	PFC	Sug	No	Clin No NFB ✗	↓ Yes	Yes	Sess	Clin ~	–	–	–
Kirschner et al., 2018	Addiction	22	# VTA and SN	Yes	Yes ✗	Healthy NFB ✓	↑ Yes	No	Yes	–	–	–	–
Zilverstand et al., 2017 $	ADHD	7	dACC	Yes	Yes ✓	* Clin Unaware ✓	↑ Yes	No	Sess	Clin ↑	–	–	ISRCTN12390961
Alegria et al., 2017, Rubia et al., 2019 $	ADHD	18	# rIFG	No	Yes ✓	* Clin Diff ROI ✓	↑ Yes	Yes	Pre-post	Clin ↑	Yes	>5m (clin ↑)	ISRCTN12800253
Buyukturkoglu et al., 2015 $	Anxiety	3	Bilateral AI	No	Yes ✓	–	↓ Yes	–	Yes	Clin ↑	–	–	–
Zilverstand et al., 2015	Anxiety	9	Insula and dlPFC	Yes	No	* Clin Unaware ✓	↓↑_diff_ Yes	Yes	Yes	Clin ↑	Yes	3 m (clin ↑)	–
Scheinost et al., 2013	Anxiety	12	OFC	Yes	Yes ✓	Clin Yoked ✗	↓↑_same_ Yes	No	Pre-post	Beh ↑	Yes	–	–
Scheinost et al., 2014 $	Anxiety	5	OFC	Yes	Yes –	Healthy NFB –	↓↑_same_ –	–	–	Clin ↑	–	–	–
Sreedharan et al., 2019, Sreedharan et al., 2020	Aphasia	4	Broca and Wernicke's areas	Sug	No	Multi. Healthy NFB and Clin No training ✓	↑ Yes	–	Sess	Beh ✗	–	–	–
Ramot et al., 2017 $	Autistic disorder	17	STS, SSC and IPL^8^	No	No	* Clin Diff con ✗	↑ Yes	Yes	Sess	Clin ✗	–	5-56w (conn ↑)	NCT01031407
Paret et al., 2016 $	BPD	10	Bilateral amygdala	No	Yes ✗	–	↓ Yes	–	Sess	Clin ✗	–	–	–
Zaehringer et al., 2019 ^	BPD	25	# Right amygdala	No	No	–	↓ Yes	–	Sess	Clin ↑	No	6w (clin ↑)	NCT02866110 DRKS00009363
Liew et al., 2016 $	Brain damage	4	M1 and ipsilateral thalamus	Sug	Yes ✓^9^	–	↑ Yes (3/4)	–	Pre-post	–	–	–	–
Sitaram et al., 2012 $	Brain damage	2	PMv	No	Yes ✓	Healthy NFB ✓	↑ Yes	–	Sess	Beh ✗^18^	–	–	–
Robineau et al., 2019 $	Brain damage	6	V1	Sug	No	Clin Diff con ✗	↑ Yes	–	Sess	Clin ↑	No	–	–
deCharms et al., 2005	Chronic pain	8	rACC	Sug	No	Multi. Clin NFB and healthy No NFB, No NFB diff str, NFB diff ROI, yoked ✓	↓↑_same_ Yes	–	Yes	Beh ↑	Yes	–	–
Guan et al., 2015	Chronic pain	8	rACC	Sug	No	** Clin Diff ROI ✗	↓↑_same_ Yes	Yes	–	Beh ↑	No	–	–
MacDuffie et al., 2018 $	Depression	13	ACC	Yes	No	Clin WS –	↓ n.a.^14^	n.a.	n.a.	–	–	–	–
Hamilton et al., 2016 $	Depression	12	Fronto-insular cortex and dACC	Yes	Yes ✓	* Clin Yoked ✓	↓ Yes	No	Pre-post	–	–	–	–
Yuan et al., 2014	Depression	14	# Left amygdala	Yes	Yes –	** Multi. Healthy NFB and Clin Diff ROI –	↑ -	–	–	Clin ↑	–	–	–
Young et al., 2014 $	Depression	14	# Left amygdala	Yes	Yes ✓	** Clin Diff ROI ✗	↑ Yes	Yes	Yes	Clin ↑	–	–	–
Young et al., 2017a, Young et al., 2017b, Young et al., 2018	Depression	19	# Left amygdala	Yes	Yes ✓	** Clin Diff ROI ✓	↑ Yes	Yes	Yes	Clin ↑	Yes	–	NCT02079610
Zotev et al., 2016 $	Depression	13	# Left amygdala	Yes	Yes ✓	** Clin Diff ROI ✗	↑ Yes	Yes	Yes	Clin ↑	–	–	–
Zotev et al., 2020 ^$	Depression	16	# Left amygdala and left rACC	Yes	Yes ✓	* Clin SHAM-C NFB ✗	↑ Yes	Yes	Yes	Clin ↑	Yes	–	–
Linden et al., 2012 $	Depression	8	Various regions: dlPFC, vlPFC, insula	No	No	Clin MR –	↑ Yes	–	Yes	Clin ↑	Yes	–	–
Peciña et al., 2018	Depression^1^	24	–	–	No	* –	n.a.	–	n.a.	–	–	–	–
Mehler et al., 2018 @	Depression	16	Mainly anterior brain areas such as insula and striatum	Sug	Yes ✗	* Clin Diff str ✓	↑ Yes	No	Sess	Clin ↑	–	18w (clin ↑)	NCT01544205
Jaeckle et al., 2019 ^@$	Depression	19	rSATL and pSCC	No	No	* Clin Diff str ✓	↓ Yes	–	Yes	Clin ↑	No	–	ISRCTN10526888
Zahn et al., 2019 $	Depression^2^	14	aSCC and aSTC	No	No	** Clin Diff con ✗	↑ Yes	Yes	Pre-post	Clin –	–	–	NCT01920490
Rance et al., 2018	Misc	10 (A), 20 (TS)^5^	OFC (A), SMA (TS)	Yes	–	** Clin Yoked –	↓↑_same_ –	–	–	Clin ↑	–	2, 4, 6, 8w (clin ↑)	NCT02206945 NCT01702077
McDonald et al., 2017	Misc	76^6^	DMN	Yes	No	–	↓↑_same_ Yes	–	– (n.a.)	–	–	–	–
Skouras and Scharnowski, 2019 @	Misc	74	DMN	Yes	No	Healthy NFB ✓	↓↑_same_ Yes	Yes	Yes	–	–	–	–
Hohenfeld et al., 2017 $	ND-A	10	PHG	Yes	No	Multi. Healthy NFB & SHAM ✓	↑ Yes	No	Yes	Clin ↑	–	–	–
Papoutsi et al., 2018b $	ND-H	10	SMA	Sug	No	–	↑ Yes	–	Sess, pre-post	Beh ✗	–	–	–
Papoutsi et al., 2018a ^$	ND-H	8 & 8^7^	SMA	Sug	Yes ✓	* Multi. Clin 2x SHAM (activity ✓ and connectivity ✗)	↑ Yes^15^	No	Yes	Beh ✗	–	3x: 2, 4–6 and 8-10w (beh & trans ✗)	–
Tinaz et al., 2018 $	ND-P	8	Right insula and dlPFC	Yes	Yes ✓	Clin No NFB –	↑ Yes	–	Yes	Clin ✗	–	–	–
Subramanian et al., 2011 $	ND-P	5	SMA	Sug	No	Clin No NFB ✓	↑ Yes	–	Yes	Clin ↑	–	2w (beh ↑)	–
Buyukturkoglu et al., 2013 $	ND-P	1	SMA	Sug	No	Healthy NFB ✓	↑ Yes	–	–	Beh ✗	–	–	–
Subramanian et al., 2016	ND-P	15	SMA	Yes	Yes ✓	Clin No NFB –	↑ Yes	–	Sess	Clin ↑	No	–	NCT01867827
Frank et al., 2012	Obesity	10	# Bilateral AI	Sug	No	Healthy NFB ✓	↑ Yes	Yes	–	–	–	–	–
Spetter et al., 2017 $	Obesity	8	dlPFC and vmPFC	Sug	No	–	↑ Yes	–	Sess	Beh ✗	–	–	–
Kohl et al., 2019	Obesity	17	Left dlPFC	No	No	* Clin Diff ROI ✓^12^	↑ Yes	No	Yes	Beh ↑	Yes	4w (beh ↑)	NCT02148770
Sitaram et al., 2014 $	PP	4	Left AI	Sug	No	–	↑ No^16^	–	Yes	Beh ~	–	–	–
Zweerings et al., 2018	PTSD	9	# ACC	Sug	Yes ✓	Healthy NFB ✓	↑ Yes	No (cont > exp)	–	Clin ↑	–	–	–
Gerin et al., 2016 $	PTSD	3	Amygdala	No	No	–	↓ Yes	–	–	Clin ↑	–	–	–
Nicholson et al., 2017	PTSD	10	Bilateral amygdala	No	Yes ✓	–	↓ Yes	–	Yes	–	–	–	–
Nicholson et al., 2018	PTSD	14	Bilateral amygdala	No	Yes ✓	–	↓ Yes	–	Yes	–	–	–	–
Misaki et al., 2018	PTSD	16	# Left amygdala	Sug	Yes –	* Multi. Clin Diff ROI and healthy veterans NFB ✓	↑ Yes	–	Yes	Clin ↑	–	–	–
Zotev et al., 2018	PTSD	15	# Left amygdala	Sug	Yes ✗	* Clin Diff ROI ✗	↑ Yes	No	Yes	Clin ↑	Yes	–	–
Cordes et al., 2015	Schizophrenia	11	# ACC	Sug	No	Healthy NFB ✓	↑ Yes	Yes	–	–	–	–	–
Dyck et al., 2016 $	Schizophrenia	3	# ACC	Sug	Yes ✓^10^	–	↑ Yes	–	Sess	Clin ~	–	–	–
Zweerings et al., 2019	Schizophrenia	21	Left IFG and left pSTG	No	No	** Healthy NFB ✓	↓↑_same_ Yes	No	–	Beh –	–	–	–
Ruiz et al., 2013	Schizophrenia	9	Bilateral AI	Sug	Yes ✗	–	↑ Yes	–	Yes	Beh ↑	Yes	–	–
Orlov et al., 2018 $	Schizophrenia	12	Left STG	No	Yes ✓	–	↓ Yes	–	Yes	Clin ✗	–	–	–
Emmert et al., 2017 $	Tinnitus	7	AC	Sug	Yes –	Clin NFB (inter ✗)	↓ Yes^17^	No	Sess	Clin ~	–	6w (clin ✗)	–
Haller et al., 2010 $	Tinnitus	6	AC	No	No	–	↓ Yes (5/6)	–	Yes	Beh ~	–	–	–

Legend per category:

*Publication:* ^ = not peer-reviewed at the time of the search; @ = sample size calculation, $ = pilot, feasibility, or proof-of-principle.

*Clinical population:* ADHD = attention deficit hyperactivity disorder; Aphasia = Expressive (Broca’s) aphasia; BPD = borderline personality disorder; PP = psychopathy; Misc = miscellaneous; ND-A = neurodegenerative disease (Alzheimer’s disease); ND-H = neurodegenerative disease (Huntington’s disease); ND-P = neurodegenerative disease (Parkinson’s disease); PTSD = post-traumatic stress disorder; ^1^ Placebo study; ^2^ The patients were remitted.

*Clinical Group Size:*^3^ The size of the experimental group (one arm only). ^4^ The article presents only the data from the experimental group (n = 22) of a previous neurofeedback study, which they split into relapsed (12) and non-relapsed group (10); ^5^ A = anxiety, TS = Tourette’s syndrome; ^6^ They performed analysis also on 121 participants, but here we report only the group of the participants that did not fall asleep; ^7^ One group received activity- and one group connectivity-based neurofeedback.

*ROI:* # anatomical localizer; ^8^ Based on a previous study.

*Strategy:* Sug = suggestions.

*Transfer run(s) and success:* tick (✓) for yes, cross (✗) for no, minus (–) for not stated; ^9^ in 2/4 participants; ^10^ in 1/3 participants.

*Control/comparison group:*^11^ Comparison group refers to healthy participants, who do not control for the effects of neurofeedback (for further explanation see the results section (item 2a)); * = single-blinded, ** = double-blinded; regulation success: tick (✓) for yes, cross (✗) for no, minus (–) for not stated; ^12^ Behavioral testing was also blinded; Clin = the same clinical population; NFB = neurofeedback; Multi = more than one control group; Diff ROI = different region of interest; diff str = different strategy; diff con = different connectivity; RM = mental rehearsal; SHAM-C = computer generated SHAM.

*Regulation Success*: ↓ = down-regulation; ↑ = up-regulation; ↓↑_diff_ = bidirectional regulation of a different region; ↓↑_same_ = bidirectional regulation of the same region; ^13^ reported in an unpublished paper; ^14^ The participants could regulate, but the regulation was not the main interest of the study; ^15^ Yes in the activity group and no in the connectivity group; ^16^ one participant could successfully regulate, but with more sessions; ^17^ The group receiving continuous feedback could regulate, the control group receiving intermittent feedback could not.

*Experimental vs Control:* cont > exp = control group significantly better regulated than the experimental group.

*Plots:* Sess = average session regulation; Pre-post = regulation before and after training; Yes = runs and session were represented; – = no plots.

*Symptom measures:* Clin = clinical measure; Beh = behavioral measure; – = not reported; ↑ = improvement; ✗ = no improvement; ~ = mixed results (some participants improved or there was an improvement over each session, but not across sessions); ^18^ Measured with TMS.

*Correlation:* Correlation between symptom improvement and regulation success.

*Follow-up:* duration between the last session and follow-up session expressed in weeks (w) or months (m); clin = clinical measure; beh = behavioral measure; trans = transfer run; conn = connectivity changes; ↑ = stable improvement; ✗ = diminished improvement.

*Registered:* the registration identifier.

#### 2.2.2 Overview of the studies

Table 2 provides an overview of the general design and results for each of the 62 studies (66 publications) investigating fMRI neurofeedback in clinical populations and can also be found in the supplementary materials. The table includes the experimental (clinical) group size, type of the localizer, targeted region of interest or connectivity network, direction of regulation, transfer runs and transfer success, and follow-up sessions with results. Due to a high variability of designs and reported results, most of the entries were classified into one of the wider categories. These are discussed under each category in the results section.

#### 2.2.3 Statistical power and sensitivity

Statistical power is the probability that an effect will be detected where it actually is present; it depends on the size of the effect and of the group (i.e., number of participants). Larger effects tend to have a higher probability (i.e., power) to be observed, and larger groups increase the probability of finding a true effect. The statistical power and sensitivity of a study design is therefore a useful indicator of the likelihood of false positives and negatives of reported results in clinical studies. It is recommended to use the calculation *a priori* in order to determine and justify the sample size needed to reliably detect a certain effect (see Table 1, item 1b of CRED-NF checklist). Since we could only evaluate the studies after they had been published, an *a priori* calculation was not suitable. We also decided not to perform a *post hoc* power analysis, as it usually only represents a *p*-value transformation and is therefore not informative (Perugini et al., 2018). Furthermore, each study defines success differently, which makes the already difficult process of defining the smallest effect size of interest (SESOI) (Lakens et al., 2018) impossible, so we decided to estimate the statistical power based on the sample sizes used, using alpha of 0.05 and the standard range of effect sizes based on Cohen’s d (i.e., d = 0.2, 0.5, and 0.8 for small, medium, and large effects, respectively) (Cohen, 1992). Note that, depending on the test used, a different value must be applied; when using ANOVA for example, Cohen’s f value needs to be used instead of Cohen’s d. Cohen’s f equals to a half of Cohen’s d, meaning the values used were 0.1, 0.25, and 0.4 (Cohen, 1988, Cohen, 1992).

Statistical sensitivity is the smallest effect size that can be detected with a given probability (i.e., power) and group size and is, compared to power estimates, in the case of the present review the more informative value as it gives an actual effect size that can be detected. Sensitivity was calculated using two probabilities: 0.8 and 0.95, and alpha of 0.05.

Both power and sensitivity were calculated for each study individually using G*Power software (Faul et al., 2007), following the approach from Kohl et al. (2020). For a calculation example please refer to Supplementary materials. For studies reporting results from a repeated-measures ANOVA no violation of sphericity and a correlation of 0.8 between repeated measures was assumed (Calamia et al., 2013). For simplicity, matching ANOVAs and t-tests were used instead of non-parametric tests; (linear) mixed models and ANOVAs with more than one factor were all treated as a two-factorial mixed ANOVA. One of the two factors included the group (if applicable). The other factor depended on the reported results; it usually included a factor used in the original model that was related to repeated measures (e.g., sessions or runs) or a condition (e.g., regulate *vs*. rest). Finally, no correction for multiple comparisons was considered and tests were performed one-sided only when so reported in the corresponding publication.

Statistical power and sensitivity were calculated for regulation and behavioral results separately. Behavioral results included only clinical outcome measures as these would be needed for a clinical trial.

Finally, a mean and a median value were determined for each power and sensitivity estimation based on the values calculated for individual studies to get a general overview of all currently available studies.

#### 2.2.4 Clinical trials

Finally, the initial 841 publication hits were scanned for registered clinical trials. Clinical trials aim to evaluate a health-related safety and/or effectiveness of an intervention (i.e., treatment) in human subjects. In order to search for information about ongoing trials beyond those already identified in the initial literature search we checked papers reporting registered trials for additional references. We then analyzed all preliminary identified papers separately to create a general overview of clinical trials, following the same procedure as described in Fig. 1. After removing duplicates found in both publications and in registries, 68 studies were found; 17 were then excluded for using a non-fMRI neurofeedback modality, and additional 10 were excluded for recruiting only healthy participants (i.e., not a clinical population). Forty-one registered clinical trials were left, including only studies of fMRI neurofeedback in clinical populations. These were then sorted based on the registration year and were, if available, matched with published results based on the trial registry number. Finally, trials were sorted based on the clinical population, following the same grouping as described for publications in section *General approach*.

## 3 Results

### 3.1 Studies and publications

**Fig. 2:**
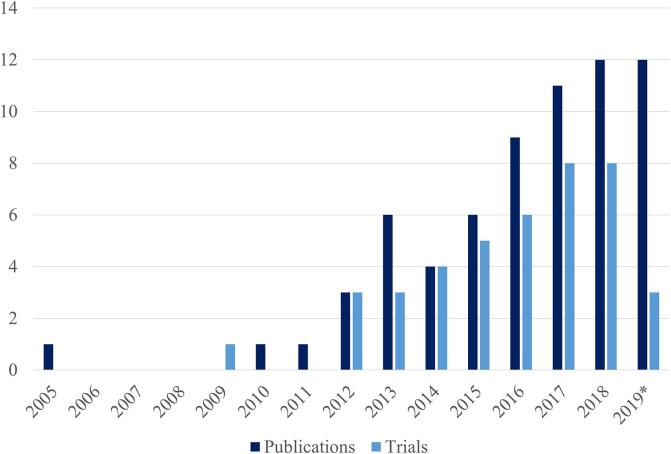
Number of published clinical papers and registered clinical trials investigating clinical populations. A steady increase of publications and registered trials is observed in the last 10 years. *Note that the count for 2019 is not complete; the final search includes studies that became available online before the 30th of October 2019.

Overall, clinical fMRI neurofeedback research has been continuously growing over the last 10 years; although the first fMRI neurofeedback study on a clinical population dates to 2005, the publication of more clinical results started 5 years later and has been steadily increasing since (Fig. 2). It is important to note that the number of publications does not necessary match the number of studies (discussed below); the search was also performed before the end of 2019, so the number of publications for 2019 is not complete. Finally, the count also includes four studies that have not (yet) been peer-reviewed; one in 2018 and three in 2019 (see Table 2).

**Fig. 3:**
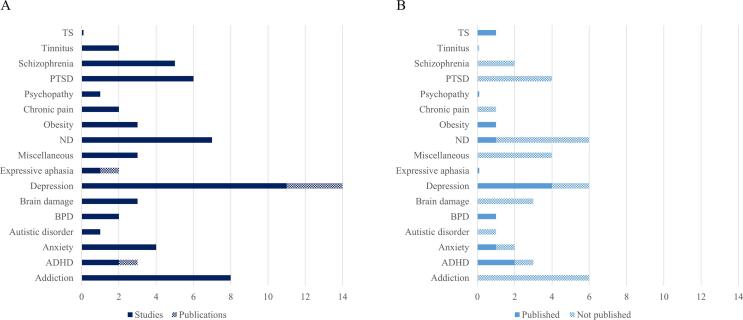
Number of neurofeedback publications and registered trials per disorder group. A) Neurofeedback studies and publications. Certain studies (dark blue) were discussed in several publications and the difference is presented in lighter shade. Depression is the most investigated disorder in the field of fMRI neurofeedback, followed by addiction and neurodegenerative disorders. B) Registered clinical trials with and without published results. Clinical trials with published results are presented in light blue. The remaining registered trials (with no published results) are presented in a lighter shade. Note that one of the studies in the miscellaneous category published results from two registered clinical trials represented under Tourette’s syndrome and anxiety. Note. TS = Tourette’s syndrome; BPD = borderline personality disorder; ADHD = attention deficit hyperactivity disorder; PTSD = post-traumatic stress disorder. Neurodegenerative diseases (ND) include Alzheimer’s, Huntington’s, and Parkinson’s disease. Brain damage includes hemineglect, hemiparalysis and stroke. Anxiety includes contamination anxiety trait of obsessive–compulsive disorder, and arachnophobia. Addiction includes alcohol, nicotine, and cocaine dependence. Miscellaneous category includes publications that investigated more than one disorder. (For interpretation of the references to colour in this figure legend, the reader is referred to the web version of this article.)

To investigate the clinical diversity, clinical populations were grouped into 16 different disorder categories, as described in *General approach* (see Fig. 3). The grouping was done due to a high variability of different applications. The highest number of publications was investigating neurofeedback effects in major depressive disorder (N_publications_ = 14, N_studies_ = 11), followed by addiction (N = 8) and neurodegenerative disorders (N = 7).

### 3.2 Clinical trials

Similar to published studies, there has been a steady increase of registered trials in clinical populations as well, starting in 2009 (see Fig. 2). 10 of 41 trials already have published results (see Table 2) and two trials have corresponding trial design publications (Cox et al., 2016, Gerchen et al., 2018). All published results of trials have a corresponding entry in one of the two international trial databases included in the search protocol. One of the published trials has also been registered in a national database. One trial design has only been registered in a national database (Gerchen et al., 2018).

Matching the registered trials with publications showed that the currently published studies were all registered between 2012 and 2016. Half of all registered trials from this period have available published results and the average timespan between the registration and publication was a bit under 4 years. In the same registration period, three additional trials have indicated that the recruitment of patients is completed and three were terminated with no results. Two trials registered after this period (both in 2017) have indicated the completed recruitment and none have been reported as terminated, meaning that the majority of the trials are currently marked as (still) in progress. Caution is however advised as trial completion statuses might not be accurately reported (Fleminger and Goldacre, 2018).

### 3.3 CRED-NF checklist

The following subsections report the conformity of all published clinical fMRI neurofeedback studies with the CRED-NF checklist. Note that the following section reports results of studies (N = 62) and not publications (see Table 2).

#### 3.3.1 Pre-register experimental protocol and planned analyses (item 1a)

Although not an *essential* item on the checklist, pre-registration of studies does help with transparency, standardization, and reproducibility, and should become fundamental in the upcoming years. It might also help fight publication bias, which could significantly alter the results presented below and give us a more accurate representation of effectiveness and clinical benefits of fMRI neurofeedback training.

Pre-registration is usually done in international online databases such as clinicaltrials.gov or International trial registry (ISRCTN) but can also be done in a national data base or even as a design proposal publication. Pre-registration in databases was reported by 10 studies (16%) and one group registered the study retrospectively. As expected, these are some of the more recent publications, with the oldest one dating back to 2016 (Subramanian et al., 2016). All pre-registered studies provided details of the protocol and also which outcome measures would be acquired and analyzed, but none of them provided a detailed statistical analysis plan.

#### 3.3.2 Justify sample size (item 1b)

Small sample sizes are one of the primary reasons for underpowered studies (Algermissen and Mehler, 2018) and yet only three studies (Jaeckle et al., 2019, Mehler et al., 2018, Skouras and Scharnowski, 2019) justified their sample size with power calculation (see Table 2). Although two studies (Papoutsi et al., 2018a, Subramanian et al., 2016) estimated the number of participants for future studies based on their early-phase results, this procedure can be problematic because it can lead to exaggerated estimates of effect sizes and thus underpowered efficacy studies. Determining the sample size using the smallest effect size related to the study’s interest, such as the minimally clinically important difference is generally more conservative (Ros et al., 2020). Furthermore, the 32 studies reported as pilot, feasibility, or proof-of-principle studies, did, by definition, not provide power calculations for clinical effects.

**Fig. 4:**
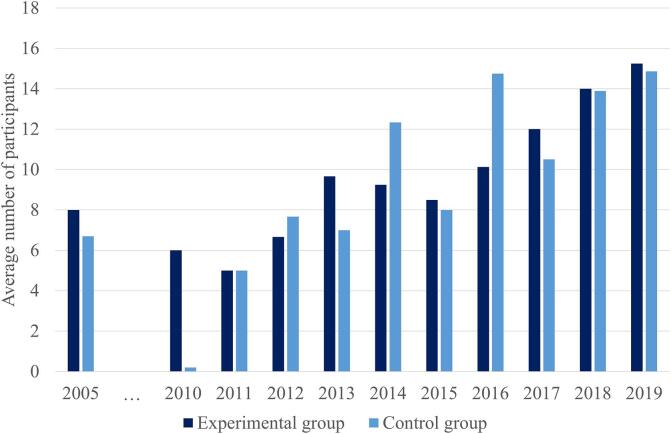
Average number of patients in the experimental and control group per year. A steady increase in the average number of recruited participants is observed. The number has doubled over the past 10 years. Note that two studies were excluded from this calculation due to much larger sample sizes (McDonald et al., 2017, Skouras and Scharnowski, 2019).

In order to see whether sample sizes indeed are increasing with more published studies, we documented sample sizes for experimental and control groups separately and then grouped each based on the year of publication. In publications with multiple control groups, an average control group size was calculated first. The two studies using a large dataset from a repository (McDonald et al., 2017, Skouras and Scharnowski, 2019) were excluded from the count as outliers. Finally, if no control group was included in the study, the study was omitted from the control group count in order to provide a realistic average control group size. This procedure indeed revealed a steady increase of an average group size per year (Fig. 4). Group sizes still show great variability per year, as additional pilot studies are performed on new clinical populations. Even though it is important to strive for bigger sample sizes, the cost of the scanning, group variability, and bias in effect size estimations, among others, question whether using sufficiently large sample sizes to increase power is indeed feasible (Boukrina et al., 2020). As for example shown by Subramanian and colleagues (Subramanian et al., 2016), 101 participant would be needed per group in their study based on their power calculation. Although this is needed to achieve sufficient power, it is difficult to achieve in practice. Regardless, even if the sample size cannot be achieved, this should be stated together with the power analysis, as recommended by CRED-NF.

#### 3.3.3 Employ control group(s) or control condition(s) (item 2a)

**Fig. 5:**
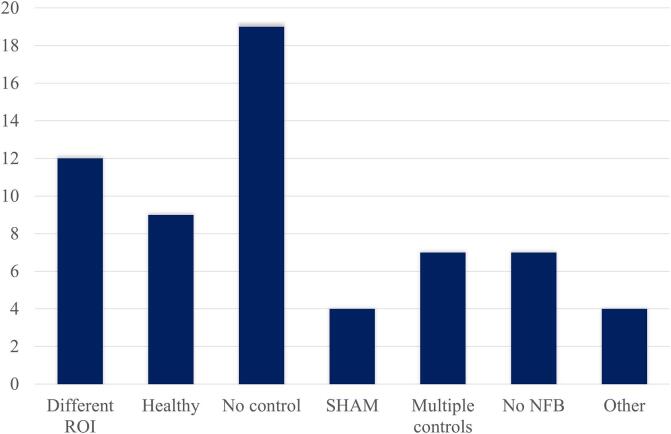
Control groups. A healthy control group performing the same task as the experimental group was used in 15% of the studies. Feedback from a different region, unrelated to the symptoms, was provided to a clinical control group in 19% of the studies. SHAM neurofeedback that was either yoked or artificially created was used in 7% of the studies. 11% of the studies did not provide feedback to the control groups. Multiple control groups were used in 11% of the studies. Finally, 31% of the studies did not use a control group.

Control groups or conditions are essential for demonstrating the neurofeedback-specific effects (for an extensive review of control groups please refer to Sorger et al. (2019)). Most clinical studies used some form of control or comparison group (69%; see Table 2 and Fig. 5). Out of these 43 studies, 27 (63%) used another clinical group with the same clinical diagnosis; clinical control groups either received the feedback from a different region (or connectivity of a different network configuration) (12/27) or received yoked (3/4) or artificially created (1/4) SHAM neurofeedback. No neurofeedback was provided to the clinical control group in 5 studies; this either meant that the participants did not receive feedback or were mentally rehearsing the task inside or outside of the scanner. Participants of two studies were unaware of the neurofeedback being presented to them on the screen. Two studies asked the control group to use a different strategy than the experimental group, one used the same group also as its control group (within subject design) and one presented a different type of feedback to the control group.

A special type of a control group are healthy participants; they are not always considered to be an actual control group (e.g., in Thibault et al. (2018)) since they perform the same task and receive actual feedback and therefore merely serve as a comparison group, but are nevertheless included in the present review for completeness. Although they pose a question of defining what exactly is a healthy participant (Marchesini et al., 2017, Pavletic, 2020) and may not in itself control for any clinical neurofeedback effects, they still do provide essential information regarding the “ideal/healthy” response or performance of the task or, in other words, confirm the effectiveness of the protocol. This is indeed important during the initial, feasibility stages of studies in order to verify that the task can be performed (e.g., the region can be controlled), but it becomes unnecessary in the later stages of trials. Healthy participants formed the comparison group in 21% of studies.

Finally, results of multiples control groups were reported in 16% of studies using control groups. These used two or more combinations of the control groups described above, most often using a combination of both a healthy and clinical group.

#### 3.3.4 When leveraging experimental designs where a double-blind is possible, use a double-blind (item 2b)

Blindness was classified as either single, double, or not blinded. Single blinding refers to either the participants or researchers (and/or clinicians) in contact with patients being unaware of the patients’ group allocation (active or control). Double blinding implies that both participants and researchers were unaware of the patient’s group allocation. A study was classified as not blinded when it clearly stated that no blinding was performed, or the publication did not mention blinding at all.

21 studies (34%) reported some sort of blinding. 13 studies (62% of all blinded) reported using single blinding and the rest used double blind designs. None of these studies reported whether the blinding was maintained.

#### 3.3.5 Report whether participants were provided with a strategy (item 3b)

In 37% of studies, participants were not provided with any strategies at all; some of these studies provided background information on the role of the targeted region or informed the participants of the direction of regulation but gave no examples of potential tasks. 34% of studies provided suggestions of strategies that might work or have worked for other participants, but let the participants choose their own. Finally, 29% of studies provided specific instructions and, in some cases, even organized a separate pre-scanning session to determine personalized strategies.

#### 3.3.6 Report neurofeedback regulation success based on the feedback signal (item 5a)

Here, successful regulation is defined as a significantly different signal in the desired direction compared to rest or baseline trials within the experimental group alone (i.e., unrelated to the comparison between the experimental and control group) and does not imply a linear change over runs or sessions. Most studies report successful regulation in patients (89%; see Table 2). Three out of these 55 studies report that only some patients successfully regulated; the rest reported significant group results. One study reported no regulation success in any patient. The rest of the studies did not report regulation results in the clinical population.

In the scope of regulation success, we also looked at the localizer type, region selection and directionality of regulation.

Localizer type refers to whether the region of interest was determined based on anatomical or functional information. When the region of interest was first anatomically selected and a subset of voxels was determined using a functional localizer, the localizer is classified as functional. Anatomical localizer was used in 24% of the studies. One study did not use a localizer (placebo study) and one used a localizer determined in a previous study. The rest (73%) used a functional localizer.

The localized regions of interest were either limited to only one specific region (or combination of regions in connectivity feedback) per study or the exact region from a larger network was determined with a functional localizer for each participant independently. The target regions were selected within the following most commonly chosen areas: anterior cingulate cortex (ACC) was regulated in 15 studies, prefrontal (PFC) and orbitofrontal (OFC) regions in 14, amygdala in 12, insula in 11, and supplementary motor area (SMA) in 6. These results include also studies where a single region from a network (e.g., ACC, PFC, or insula) was selected for each participant.

We also investigated the directionality of regulation. In 55% of the studies, patients were instructed to up-regulate the region of interest. Down-regulation was expected in 26% of the studies. Finally, 16% of the studies asked the patients to regulate bi-directionally, either within the same region (N = 8) or in different regions (N = 2). Two studies provided no information regarding regulation directionality.

#### 3.3.7 Plot within-session and between-session regulation blocks of feedback variable(s), as well as pre-to-post resting baselines or contrasts (item 5b)

Visual representations can often simplify results and make them more comprehensive. However, the clinical studies summarized in this review tend to use them to cover fewer results than are reported in the text. That being said, most studies still provided plots for at least one part of results of at least the experimental group (73%); a third of these 45 studies provided plots only for session comparisons (average session results), 13% provided visualizations only for pre-to-post results, and the rest provided results for all the runs (53%).

Seven out of remaining 17 studies did not report any regulation results that could be visualized.

#### 3.3.8 Statistically compare the experimental condition/group to the control condition(s)/group(s) (not only each group to the baseline measures) (item 5c)

The comparison of the experimental and control group was reported in Table 2 if a significance test was stated; descriptive comparisons were excluded. More than half of the studies (56%) did not report any statistical comparison between the experimental and control group. Of the remaining 27 studies, 13 (48%) reported the experimental group to be significantly better at regulation, 12 (44%) reported no difference between the groups, and two reported the control group to be better at regulating.

In order to understand why so few studies reported the group comparison, we first looked at the regulation success reports of only the control group; 44% of studies did not report any regulation results. This percentage closely matches the number of studies not using a control group or not using neurofeedback in the control group, meaning that most studies using a control group also reported their results. Crucially, however, this still indicates that many studies with a control group did not directly compare the two (or more) groups.

Out of 35 studies reporting the regulation success of the control group, 66% reported that the control group could regulate their brain activity. One of these studies reported success in the activation feedback control group, but not the connectivity control group. The control groups in the rest of the studies could not regulate.

#### 3.3.9 Include measures of clinical or behavioral significance, defined a priori, and describe whether they were reached (item 6a)

**Fig. 6:**
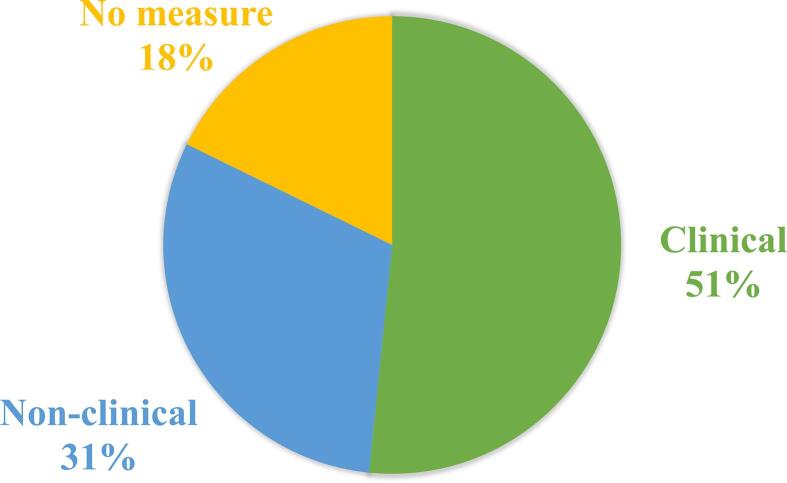
Clinical and behavioral improvement measurements. Most studies measured symptom improvement with clinical questionnaires. The rest used behavioral measures or introspective reports of the participants. 18% of the studies did not report any measures of improvement.

To measure clinical or behavioral significance, different questionnaires, behavioral tests, or subjective reports can be used. For the purpose of this review, only measures performed both before and after neurofeedback training were reported in Table 2. As seen in Fig. 6, most studies included at least one clinical or behavioral measurement (82%).

Improvement of symptoms reported in Table 2 was defined as such when at least one of the clinical or behavioral measures showed significant differences in results before and after the neurofeedback training in the experimental group. These differences were mostly statistically significant, but some studies using clinical measures reported also clinically significant improvements. Out of 78% of studies reporting results, 60% (29/48) reported significant improvement of symptoms, 27% reported no difference, and the remaining 6 (13%) reported some improvement (descriptively in at least some participants or statistically within, but not across sessions).

#### 3.3.10 Run correlational analyses between regulation success and behavioral outcomes (item 6b)

Correlation analysis was reported in Table 2 when it was performed between regulation success and behavioral change (pre-to-post). The majority of the studies (72%) did not report correlation test results between the regulation success and clinical improvement. A significant correlation was found in 65% (11/17) of the remaining studies. In the rest, the correlation was not significant.

#### 3.3.11 Other findings

We also looked into some other design components that are relevant when discussing clinical benefits of neurofeedback, namely transfer runs and follow-up sessions.

Transfer runs are important to show that the strategies, learnt during neurofeedback training, can also be used beyond the training sessions. If the targeted symptom for example included auditory hallucinations, it is essential that patients can successfully use the learnt strategies in daily life to decrease the disturbing effects of hallucinations.

Transfer runs were utilized in 47% of the studies and transfer results were reported in 86% (25/29) of these studies (Table 2). Transfer runs were used either at the end of each neurofeedback session, at the end of the last neurofeedback session, or during a separate session, usually a few days after the last training session. Participants were instructed to regulate their brain activity in the same way as during neurofeedback runs but they received no neurofeedback information. Transfer success is here defined in the same way as regulation success, but without neurofeedback: a significantly different activation in the desired direction during regulating trials compared to baseline or other contrasting condition. Success was shown in 80% (N = 20) of the studies with a transfer run. Two of these studies reported that only individual participants achieved significant transfer success.

Follow-up sessions were defined as separate sessions which were performed at least a few weeks after the last neurofeedback session and included either behavioral or regulation testing results. Behavioral post-sessions were not treated as a follow-up if the last neurofeedback session did not include the same tests. Follow-up sessions are an essential component of neurofeedback research as they investigate long-lasting effects of neurofeedback. In other words, they serve to check whether the potential clinical improvement in patients diminished and whether the participants remitted.

Ten studies (16%) reported using a follow-up session, scheduled anytime between 2 and 56 weeks after the last regulation session. One study asked the patients to perform additional two neurofeedback runs, one instructed them to regulate without neurofeedback, and one tested the resting state connectivity changes. The rest measured clinical and behavioral changes. Ideally, studies should measure at least clinical or behavioral changes to investigate long-term effects of regulation training on behavioral outcomes.

Eight out of ten studies reported retained (or even further enhanced) improvement, mostly by comparing the follow-up to the pre-test or first session results. Two studies reported no significant difference between the follow-up and baseline scores, meaning the symptoms returned to the pre-neurofeedback severity. Interestingly, the time duration between the last session and the follow-up session did not seem to influence the results as the studies with the longest durations all reported maintained positive effects.

### 3.4 Statistical power and sensitivity

#### 3.4.1 Regulation success

32 studies were reported as pilot, feasibility, or proof-of-principle studies. These studies usually use small sample sizes and do not require an *a priori* power calculation and should therefore also not be performing inferential statistical tests (Lancaster et al., 2004). We therefore extracted the number of participants per group only for the remaining 26 studies that performed a group analysis. Most studies included between 4 and 35 participants per group; the two studies that used datasets from a repository included much larger groups of 76 participants in the first study and 62 and 74 participants per group in the other study. The group size variability provided a vast range of estimates (estimation of detected effect size with a power of 95% for example ranging from 0.20 to 2.65; for values per study please refer to supplementary materials). Both the mean and the median values were therefore calculated (see Table 3) and only median results are reported here.

**Table 3:** Statistical power and sensitivity for regulation success and clinical measures excluding the pilot, proof-of-principle, and feasibility studies. The mean and median values of statistical power and sensitivity are presented, but only for studies that were not labeled as pilot, feasibility, or proof-of-principle. Power is estimated (in percentage) for small, medium, and large effects (based on Cohen’s d of 0.2, 0.5, and 0.8, respectively). Sensitivity, or estimation of detected effect size (based on Cohen’s d) with a certain power, is calculated for the power of 80 and 95%. Power and sensitivity calculations for regulation success include studies reporting a group analysis; clinical measures include power and sensitivity estimation for studies performing a group analysis of clinical measures. For additional information, including individual values for each study, see the Supplementary materials.

			Power			Sensitivity (in Cohen’s d)	
		N	d = 0.2	d = 0.5	d = 0.8	Power = 80%	Power = 95%
Regulation success	Mean	29.88	0.24	0.61	0.76	0.77	>0.99
	Median	22.50	0.15	0.67	0.98	0.58	0.73

Clinical measures	Mean	26.73	0.31	0.73	0.85	0.58	0.74
	Median	27	0.30	0.98	>0.99	0.36	0.46

A median sample size of 22.5 participants was used for the analysis. Median power to detect small effects only reached 15%; power for medium effects was 67%. Large effects however reached a median power of 98%, showing that they can reliably be detected. Indeed, with 80% and 95% power, effect sizes larger than 0.58 and 0.73, respectively, could be detected, confirming that, depending on the chosen statistical test, large, but also medium effects can be detected. The present studies are underpowered to detect small effects.

Given that we excluded almost half of the studies, we nevertheless calculated statistical power and sensitivity also for all 51 studies performing a group analysis combined (results are presented in the supplementary materials). The power and sensitivity estimations stayed almost identical, even though the median value of sample size decreased to 18 (as seen in the supplementary materials).

#### 3.4.2 Clinical measures

11 studies were not marked as pilot, feasibility, and proof-of-principle, but performed a group analysis and provided sufficient information to estimate statistical power and sensitivity. A median sample size of 27 participants was used for the analysis.

Here, both medium and large effects could be detected with high power (98% and >99.9%, respectively), but not small effects (with a power of 30%). When calculating the effect sizes that could be reliably detected with the power of 80 and 95%, effect sizes of 0.36 and 0.46, respectively, were estimated, confirming that medium and large effects can be detected and showing on the descriptive level that smaller effects could be detected for behavioral effects compared to regulation success.

Lastly, we calculated the sensitivity and power for clinical measures for all 27 studies that performed a group analysis of clinical measures, including the ones marked as pilot, feasibility, and proof-of-principle (results reported in supplementary materials). Thirty-three studies reported using clinical scales or questionnaires. Out of these, 27 (82%) reported a group analysis and provided sufficient information to estimate statistical power and sensitivity. The median sample size of the experimental group was, as expected, smaller: 22 participants. Unlike for regulation success estimates, the sensitivity values for clinical measures increased, meaning medium effects could be detected (median of 0.50 and 0.63 for the power of 80% and 95%, respectively, instead of 0.36 and 0.46, respectively).

## 4 Discussion

The main goal of this review was to investigate the reporting consistency of the methods and results in clinical fMRI neurofeedback studies. This investigation is an important precondition for an evaluation of the evidence for clinical benefits of fMRI neurofeedback. The main finding of this review is that the field is currently extremely diverse, investigating neurofeedback effects in many different disorders, but with small sample sizes, limited reports of certain crucial measures, little standardization, and statistical power to detect middle and large, but not small effects.

### 4.1 Quality of reporting

So far, the conclusions of currently available reports tend to describe their results as promising regardless of the level or type of performance, considering both neural and clinical results. 89% of studies reported successful regulation (e.g., task *vs*. baseline) and 57% of studies reported any type of clinical or behavioral improvement in at least some participants. However, only 28% of studies investigated if there was any relation between regulation performance and clinical/behavioral outcomes. Much of the reviewed literature lacked a clear distinction between successful self-regulation, improvement of symptoms, and evidence of a relationship between regulation and symptom improvement.

Reassuringly, most studies already report regulation success (90%) and employ some type of a control measure (69%), trying to showcase the region specificity of neurofeedback and ensuring that the positive regulation and clinical results are not due to placebo effects. Multiple reasonably sized control groups are almost impossible to utilize due to time, scanning costs and *post hoc* corrections for multiple comparisons, which would further increase the necessary sample sizes, but proper statistical comparisons within and between the groups are vital, whenever possible. Only 37% of the studies with a control group however reported a comparison between the regulation success of the experimental and control groups. When a control group is included, but no regulation group comparison is performed, it is hard to estimate whether the experimental group outperformed the control group, or in other words, show that neurofeedback really is crucial for improving self-regulation. It is worth noting that, depending on the test used, the comparison could potentially change the power calculation for those studies, as well as the required number of participants.

Furthermore, a potential issue with control groups also arises when discussing blinding. Although no studies reported checking whether the blinding was maintained, this does not necessarily mean that the blinding was checked or maintained; if it is not kept, control participants can perform worse due to lower motivation, which makes it hard to interpret the cause of potential performance and clinical differences between groups. Control groups often receive feedback that does not represent their actual performance (9% out of all control groups) or originates from a region unrelated to the task performed and the targeted clinical symptoms (28%); such a region is particularly hard to define because it should have similar properties (e.g., ease of control) to the region-of-interest. Making sure that participants in all groups retain the same level of motivation and stay blinded to their group assignment is therefore an important part of each study that should be controlled and reported.

Another important point to address are clinical or behavioral results. Although they are reported relatively often (82%) and many of these studies (73%) already provide some evidence of clinical improvement, the chosen measures tend to be relatively diverse. A universal test for clinical benefits of neurofeedback would of course be very difficult or even impossible to establish but being in accord with standard test batteries for each disorder or symptoms would allow for an easier comparison of results and would be more informative of the neurofeedback benefits. A good example can be seen in Parkinson’s disease; three out of four studies reported clinical measures and they all used Unified Parkinson’s Disease Rating Scale (UPDRS), allowing the comparison of clinical results also across the studies.

What is currently severely lacking, however, is a uniform way of showing a relation between regulation success and clinical improvement. Some studies correlated initial clinical scores with the regulation success, which might be useful to reveal differences between performers and non-performers, but only 28% of studies reported calculating a correlation between the actual change of the two measures. In order to showcase the necessity for neurofeedback, symptom reduction should reliably be associated with neurofeedback, and not with other variables, such as time and other treatments. However, the question remains if the correlation provides the full picture and should be used as the main indicator of effectiveness; 35% of the studies that calculated a correlation did not find a significant relation between the regulation and clinical improvement. Only one study of these studies reported no clinical improvement. What caused the improvement in the rest? It is worth considering that neurofeedback might be effective even when the correlation is not significant, but the group receiving neurofeedback clearly shows a clinical benefit.

Lastly, the sample sizes tend to be low. Although more than half of the studies (52%) were described as pilot, feasibility, or proof-of-principle, this was not always clear. Publications should avoid mentioning the nature of their study merely in the limitation section of their discussion; instead, the type of the study should ideally be clearly indicated in the title, as indicated by the CONSORT guidelines (Eldridge et al., 2016). This also serves as a justification of their sample size.

### 4.2 Effects of neurofeedback training

Besides reporting results, reporting analysis steps is also essential. Although CRED-NF focuses on online processing of the data (item 3d and 4d), attention should also be given to offline analysis. A number of studies did not sufficiently report their analysis plans, so some statistical tests had to be inferred (10 out of 82) based on reported results or common knowledge of statistics. The estimations, calculated in this review, included sample sizes, liberal assumptions (no multiple comparison correction, high correlations between repeated measures) and (mostly) simplified statistical tests instead of actual outcome measures, which could also potentially inflate the estimated power. A recent meta-analysis of fMRI neurofeedback however found very similar results, namely medium effect sizes (Hedges’ g = 0.59) for regulation and medium-to-small (g = 0.37) for clinical measures with 95% confidence interval (Dudek and Dodell-Feder, 2020), suggesting that our estimations do not seem to be too liberal.

Although the results in the clinical fMRI neurofeedback field tend to be overstated, the estimated (median) sensitivity still shows that medium to large effects can be reliably detected, which can be regarded as a very encouraging result. One might however want to be cautious when making conclusions regarding the detected effect sizes, considering big differences between mean and median results. This can be attributed to a large range of sample sizes and corresponding outliers, not only in pilot, feasibility, and proof-of-principle studies, which in their nature do not require power calculations and usually consist of small sample sizes, but also in the rest.

Due to the predominantly small sample sizes of the existing fMRI neurofeedback trials, it seems that these were simply underpowered to reveal clinical effects of small sizes, as are commonly expected for new add-on treatments. Both favorable and unfavorable results should always be reported in order to get a realistic judgement of potential benefits.

Not only are the sample sizes small, but there are still only a limited number of studies published per clinical population, which challenges any attempt of drawing conclusions regarding clinical success in each population. Neurofeedback in depression, for example, currently seems to provide the most complete and compelling evidence of its benefits with 12 studies, but even the smallest effects were obtained in small patient samples (up to 24 per group), which makes it hard to generalize these findings to an entire population. Considering a 2x2 mixed ANOVA, where one would compare an experimental and control group before and after treatment and would expect a significant interaction with alpha of 0.05 and the power of 80%, the study would still need 82 participants in total to detect small effects, which is much more than currently reported. Furthermore, considering a two-tailed *t*-test to compare the two groups, the sample size rises to 394 participants per group.

However, it is worth remembering that neurofeedback as a potential clinical tool is intended to treat individuals. The desired outcome in single patients is not to have small, but at least moderate effects in symptom improvement. First, feasibility of using fMRI neurofeedback in a certain clinical population should be, and in some cases already is, demonstrated. Next, studies with large sample sizes are required for stratification, i.e., dividing a clinical population into subpopulations based on certain traits or symptoms. Although so far unsuccessful (Haugg et al., 2020, Weber et al., 2020), partly due to small sample sizes and small number of studies per clinical population, the increasing data and knowledge of the existing and future studies could hopefully be used to identify subpopulations that are successful responders in the future. Although neuroimaging studies usually heavily rely on group results, individual results might prove to be informative as well, in order to estimate how many participants respond to treatment, and furthermore, to extract any characteristics of potential responders. Once stratified, subpopulations with large effects can hopefully be effectively treated.

## 5 Conclusion

fMRI neurofeedback is still a young field, but with promising currently available results that have the potential to influence future treatment alternatives, if it can be shown that the costs and demand for experts and resources are justified compared to other available treatments. In order to achieve this, the field needs to strive for more consistency and uniformity in reporting basic information, but this does not mean that additional analysis steps and results need to be omitted; on the contrary, any additional information is encouraged. Following guidelines, such as the CRED-NF checklist, would be a good first step towards standardizing the currently employed methods and results reporting to enable more accurate conclusions regarding fMRI neurofeedback benefits.

## Funding

This work was supported by the European Commission’s Health Cooperation Work Programme of the 7th Framework Programme, under the Grant Agreement n° 602186 (BRAINTRAIN).

## CRediT authorship contribution statement

**Anita Tursic:** Conceptualization, Data curation, Formal analysis, Investigation, Writing - original draft, Writing - review & editing. **Judith Eck:** Conceptualization, Data curation, Investigation, Writing - review & editing. **Michael Lührs:** Conceptualization, Writing - review & editing. **David E.J. Linden:** Conceptualization, Supervision, Writing - review & editing. **Rainer Goebel:** Conceptualization, Funding acquisition, Supervision, Writing - review & editing.

## Declaration of Competing Interest

The authors declare the following financial interests/personal relationships which may be considered as potential competing interests: Anita Tursic, Judith Eck, Michael Lührs and Rainer Goebel are employed by the research company Brain Innovation B.V., Maastricht, The Netherlands. Other authors have nothing to declare.
