# Supplementary material for: A systematic review of fMRI neurofeedback reporting and effects in clinical populations
Source: Neuroimage Clin. 2020 Nov 11;28:102496. doi: 10.1016/j.nicl.2020.102496 (PMC7724376; doi:10.1016/j.nicl.2020.102496)
Supplement: Supplementary data 1 [file mmc1.docx]

**Statistical power and sensitivity calculation example**

Statistical power and sensitivity estimations were calculated using the G*Power software and were based on the sample sizes used in each study, using alpha of 0.05. Sensitivity was calculated using two probabilities: 0.8 and 0.95. Effect sizes were estimated using the standard range of effect sizes based on Cohen’s d (i.e., d = 0.2, 0.5, and 0.8 for small, medium, and large effects, respectively) (Cohen, 1992). For studies reporting results from a repeated-measures ANOVA no violation of sphericity and a correlation of 0.8 between repeated measures was assumed (Calamia et al., 2013).

The following example (using the information provided by (Alegria et al., 2017; Rubia et al., 2019)) shows how the calculation is performed per study. The calculation was performed using a 2x2 mixed ANOVA, using the sample size of 31.

**
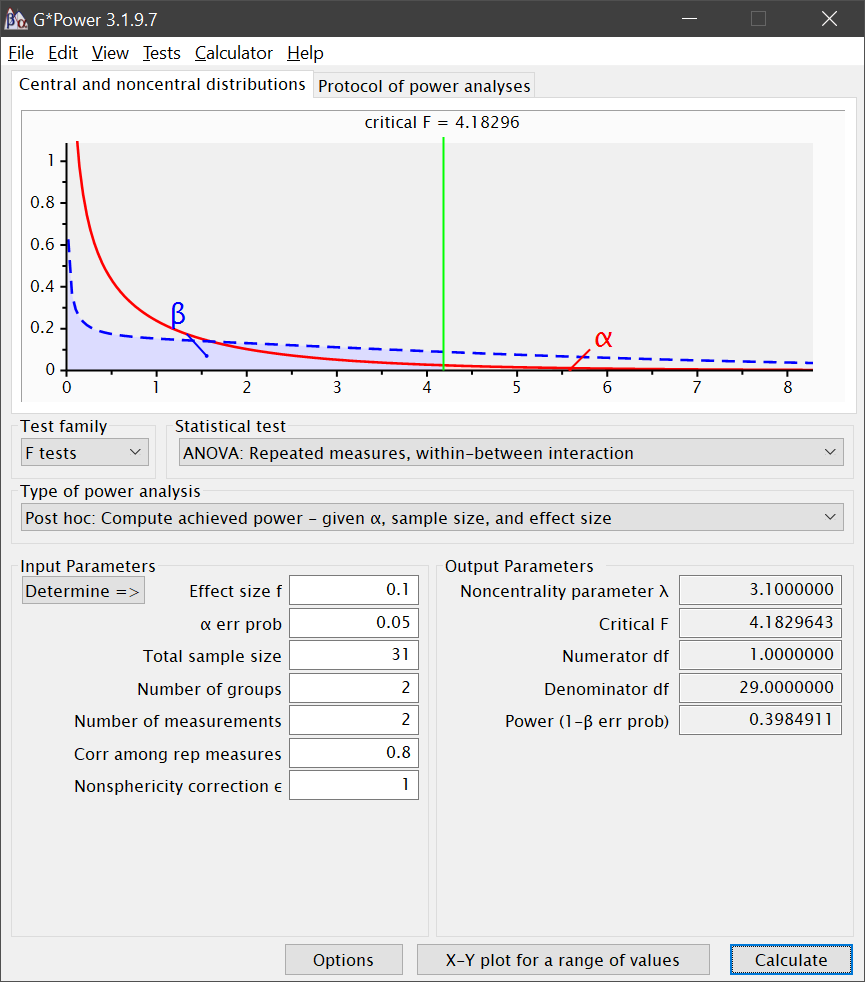
**

Figure S1. An example of power calculation for small effects using a 2x2 mixed ANOVA in G*Power.

As seen in figure S1, for power analysis, the “ANOVA: Repeated measures, within-between interaction” statistical test was used, and the selected type of power analysis was “Post hoc: Compute achieved power”. We first performed the calculation for a small effect size. Note that, when using ANOVA, Cohen’s f value needs to be used as an input in G*Power. Cohen’s f equals to a half of Cohen’s d, meaning the value used was 0.1 (Cohen, 1988). Alpha was kept at 0.05, sample size was set to 31, number of groups and number of measures were both set to 2, correlation among repeated measure was set to 0.8, and finally, no sphericity violation was assumed, so the value was kept at 1. The result is seen as the last output parameter, named Power, and equals to 0.398.

The same procedure was repeated for medium (f=0.25) and large (f=0.4) effect sizes.

Sensitivity was calculated similarly (see figure S2), using the “Sensitivity: Compute achieved power” type of power analysis. Instead of the effect size, the power of 0.8 was entered. The rest of the parameters stayed the same as during the power calculation. The calculated effect size equals to 0.165, but be aware that the obtained value corresponds to Cohen’s f. In order to compare this value to the values of other tests, it first needs to be transformed into Cohen’s d by doubling it (d=0.33).

The same procedure was then followed also for the power of 0.95.


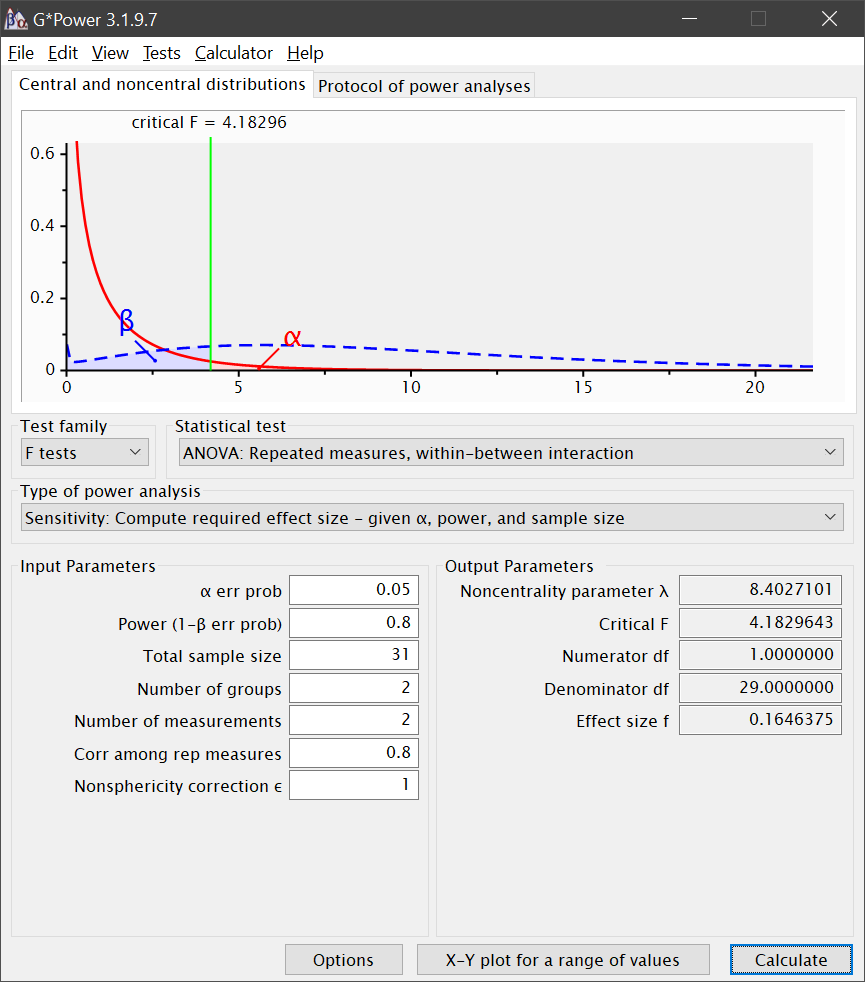


Figure S2. An example of sensitivity calculation for power of 0.8 using a 2x2 mixed ANOVA in G*Power.

**Table S1**

***Statistical power and sensitivity for regulation success of individual studies.***

*Power is estimated (in percentage) for small, medium, and large effects (based on Cohen’s d of 0.2, 0.5, and 0.8, respectively). Sensitivity, or estimation of detected effect size (based on Cohen’s d) with a certain power, is calculated for the power of 80 and 95%. Power and sensitivity calculations for regulation success include all studies that performed a group analysis and reported sufficient information. Two calculations are presented at the bottom of the table: the results in black exclude pilot, proof-of-principle, and feasibility studies (N=26); the results in grey include all studies that performed a group analysis (N=51). Pilot, feasibility, and proof-of-principle studies are marked in grey.*

| \|  \|  \|  \|  \|  \| **Power** \| \| \|  \| **Sensitivity (in Cohen’s d)** \| \| \| --- \| --- \| --- \| --- \| --- \| --- \| --- \| --- \| --- \| --- \| --- \| \| **Publication** \| **Test** \| **Criterion** \| **N** \|  \| **d = 0.2** \| **d = 0.5** \| **d = 0.8** \|  \| **Power = 80%** \| **Power = 95%** \| \| (Canterberry et al., 2013) \| 2x3 mixed ANOVA \| Condition (crave vs regulate) X Visit (3 visits) \| 9 \|  \| 0.14 \| 0.64 \| 0.97 \|  \| 0.60 \| 0.76 \| \| (Li et al., 2013) \| RM ANOVA \| Four time points \| 12 \|  \| 0.20 \| 0.88 \| 1.00 \|  \| 0.45 \| 0.57 \| \| (Karch et al., 2015) \| 2x5 mixed ANOVA \| Group X Time (5 time points) \| 27 \|  \| 0.50 \| 1.00 \| 1.00 \|  \| 0.27 \| 0.34 \| \| (Kim, Yoo, Tegethoff, Meinlschmidt, & Lee, 2015) \| Independent t-test* \| Group difference \| 14 \|  \| 0.06 \| 0.14 \| 0.28 \|  \| 1.63 \| 2.10 \| \| (Hartwell et al., 2016) \| 2x3 mixed ANOVA \| Group X Visit (3 visits) \| 44 \|  \| 0.61 \| 1.00 \| 1.00 \|  \| 0.25 \| 0.31 \| \| (Kirschner et al., 2018) \| 2x4 mixed ANOVA \| Group X Run (4 runs) \| 50 \|  \| 0.75 \| 1.00 \| 1.00 \|  \| 0.21 \| 0.27 \| \| (Zilverstand et al., 2017) \| 2x4 mixed ANOVA \| Group X Session (4 sessions) \| 13 \|  \| 0.22 \| 0.91 \| 1.00 \|  \| 0.43 \| 0.55 \| \| (Alegria et al., 2017; Rubia et al., 2019) \| 2x2 mixed ANOVA \| Group X ROI \| 31 \|  \| 0.40 \| 0.99 \| 1.00 \|  \| 0.33 \| 0.42 \| \| (Zilverstand et al., 2015) \| 2x2 mixed ANOVA \| Group X Task (regulate vs watch) \| 18 \|  \| 0.24 \| 0.88 \| 1.00 \|  \| 0.44 \| 0.57 \| \| (Scheinost et al., 2013) \| Independent t-test \| Group difference \| 23 \|  \| 0.07 \| 0.21 \| 0.45 \|  \| 1.23 \| 1.58 \| \| (Sreedharan, Arun, Sylaja, Kesavadas, & Sitaram, 2019; Sreedharan, Chandran, et al., 2019) \| Independent t-test \| Group difference \| 8 \|  \| 0.08 \| 0.16 \| 0.26 \|  \| 2.00 \| 2.65 \| \| (Ramot et al., 2017) \| Independent t-test \| Group difference \| 27 \|  \| 0.08 \| 0.23 \| 0.49 \|  \| 1.16 \| 1.50 \| \| (Paret et al., 2016) \| One sample t-test \| Condition difference \| 10 \|  \| 0.09 \| 0.29 \| 0.62 \|  \| 1.00 \| 1.29 \| \| (Zaehringer et al., 2019) \| 2x4 mixed ANOVA \| Condition (regulate vs view) X Time (4 sessions) \| 25 \|  \| 0.42 \| 1.00 \| 1.00 \|  \| 0.30 \| 0.38 \| \| (Liew et al., 2016) \| Paired t-test \| “Early” vs “late training” \| 4 \|  \| 0.06 \| 0.11 \| 0.21 \|  \| 2.13 \| 2.83 \| \| (Sitaram et al., 2012) \| Paired t-test^#^ \| Third vs first session \| 6 \|  \| 0.07 \| 0.17 \| 0.36 \|  \| 1.43 \| 1.87 \| \| (Robineau et al., 2019) \| RM ANOVA \| 3 visits \| 6 \|  \| 0.10 \| 0.43 \| 0.83 \|  \| 0.77 \| 0.97 \| \| (Hamilton et al., 2016) \| Paired t-test (one-tailed) \| Group difference \| 22 \|  \| 0.23 \| 0.73 \| 0.98 \|  \| 0.55 \| 0.73 \| \| (Yuan et al., 2014) \| Independent t-test \| Group difference \| 27 \|  \| 0.08 \| 0.24 \| 0.51 \|  \| 1.12 \| 1.45 \| \| (Young et al., 2014) \| 2x3 mixed ANOVA \| Group x ROI (3) \| 21 \|  \| 0.31 \| 0.98 \| 1.00 \|  \| 0.36 \| 0.46 \| \| (Young, Misaki, et al., 2017; Young et al., 2018; Young, Siegle, et al., 2017) \| 2x2 mixed ANOVA \| Group X ROI \| 36 \|  \| 0.45 \| 1.00 \| 1.00 \|  \| 0.15 \| 0.20 \| \| (Zotev et al., 2016) \| 2x4 mixed ANOVA \| Group X Time (4 time points) \| 24 \|  \| 0.40 \| 1.00 \| 1.00 \|  \| 0.31 \| 0.39 \| \| (Zotev et al., 2019) \| Independent t-test \| Group difference \| 24 \|  \| 0.07 \| 0.20 \| 0.42 \|  \| 1.27 \| 1.63 \| \| (Linden et al., 2012) \| 4x3 mixed ANOVA^#^ \| Session (4) X Run (3) \| 8 \|  \| 0.12 \| 0.51 \| 0.90 \|  \| 0.69 \| 0.88 \| \| (Mehler et al., 2018) \| 2x5 mixed ANOVA \| Group X Session (5) \| 32 \|  \| 0.59 \| 1.00 \| 1.00 \|  \| 0.25 \| 0.31 \| \| (Jaeckle et al., 2019) \| 2x2 mixed ANOVA^#^ \| Time X Condition \| 19 \|  \| 0.26 \| 0.90 \| 1.00 \|  \| 0.43 \| 0.56 \| \| (Zahn et al., 2019) \| 2x2 mixed ANOVA \| Group X Condition (Guilt, indignation) \| 28 \|  \| 0.36 \| 0.98 \| 1.00 \|  \| 0.35 \| 0.45 \| \| (McDonald et al., 2017) \| Correlation \| Correlation with ideal time course \| 76 \|  \| 0.41 \| 1.00 \| 1.00 \|  \| 0.31 \| 0.40 \| \| (Skouras & Scharnowski, 2019) \| Independent t-test \| Group difference \| 136 \|  \| 0.21 \| 0.82 \| 1.00 \|  \| 0.49 \| 0.63 \| \| (Hohenfeld et al., 2017) \| 3x9 mixed ANOVA* \| Group (3) X Run (9) (run) \| 30 \|  \| 0.57 \| 1.00 \| 1.00 \|  \| 0.25 \| 0.30 \| \| (Papoutsi, Weiskopf, et al., 2018) \| 4x4 mixed ANOVA \| Visit X Run \| 10 \|  \| 0.10 \| 0.47 \| 0.92 \|  \| 0.52 \| 0.66 \| \| (Papoutsi, Magerkurth, et al., 2018) \| 2x4 mixed ANOVA \| Group (treatment vs control) X Session \| 16 \|  \| 0.27 \| 0.96 \| 1.00 \|  \| 0.39 \| 0.49 \| \| (Tinaz et al., 2018) \| Paired t-test^#^ \| Difference between the before and after control run (no NFB) \| 8 \|  \| 0.08 \| 0.23 \| 0.50 \|  \| 1.16 \| 1.50 \| \| (Subramanian et al., 2011) \| One sample t-test^#^ \| Condition difference \| 5 \|  \| 0.06 \| 0.14 \| 0.28 \|  \| 1.68 \| 2.20 \| \| (Subramanian et al., 2016) \| One sample t-test^#^ \| Condition difference \| 15 \|  \| 0.11 \| 0.44 \| 0.82 \|  \| 0.78 \| 1.00 \| \| (Frank et al., 2012) \| Independent t-test* \| Group difference \| 21 \|  \| 0.07 \| 0.19 \| 0.41 \|  \| 1.29 \| 1.66 \| \| (Spetter et al., 2017) \| 3x4 mixed ANOVA \| Run (3) X Session (4) \| 8 \|  \| 0.10 \| 0.46 \| 0.46 \|  \| 0.71 \| 0.88 \| \| (Kohl, Veit, et al., 2019) \| 2x2 mixed ANOVA \| Group X Condition \| 36 \|  \| 0.45 \| 1.00 \| 1.00 \|  \| 0.30 \| 0.39 \| \| (DeCharms et al., 2005) \| Paired t-test^#^ \| First to last run \| 8 \|  \| 0.08 \| 0.23 \| 0.50 \|  \| 1.16 \| 1.50 \| \| (Guan et al., 2015) \| Independent t-test (one-tailed) \| Group difference \| 14 \|  \| 0.10 \| 0.22 \| 0.40 \|  \| 1.43 \| 1.89 \| \| (Zweerings et al., 2018) \| Independent t-test* \| Group difference \| 18 \|  \| 0.07 \| 0.17 \| 0.36 \|  \| 1.41 \| 1.81 \| \| (Nicholson et al., 2017) \| 2x3 mixed ANOVA \| Condition (regulate vs view) X Run (3) \| 10 \|  \| 0.16 \| 0.70 \| 0.98 \|  \| 0.56 \| 0.71 \| \| (Nicholson et al., 2018) \| 2x3 mixed ANOVA \| Condition (regulate vs view) X Run (3) \| 14 \|  \| 0.21 \| 0.87 \| 1.00 \|  \| 0.46 \| 0.58 \| \| (Misaki et al., 2018) \| 3x2 mixed ANOVA \| Group (3) X Session (baseline, post-training) \| 33 \|  \| 0.32 \| 0.98 \| 1.00 \|  \| 0.36 \| 0.46 \| \| (Zotev et al., 2018b) \| 2x4 mixed ANOVA \| Group X Run (4) \| 23 \|  \| 0.39 \| 1.00 \| 1.00 \|  \| 0.32 \| 0.40 \| \| (Cordes et al., 2015) \| Independent t-test \| Group difference \| 22 \|  \| 0.07 \| 0.20 \| 0.43 \|  \| 1.26 \| 1.62 \| \| (Zweerings et al., 2019) \| Independent t-test \| Group difference \| 56 \|  \| 0.11 \| 0.43 \| 0.81 \|  \| 0.79 \| 1.01 \| \| (Ruiz et al., 2013) \| Paired t-test \| Condition difference \| 9 \|  \| 0.08 \| 0.26 \| 0.56 \|  \| 1.07 \| 1.38 \| \| (Orlov et al., 2018) \| 3x2 mixed ANOVA \| Session (3) X Run (first, last). \| 12 \|  \| 0.18 \| 0.80 \| 1.00 \|  \| 0.50 \| 0.63 \| \| (Emmert et al., 2017) \| 2x3 mixed ANOVA \| Group X Session (3) \| 14 \|  \| 0.21 \| 0.87 \| 1.00 \|  \| 0.46 \| 0.58 \| \| (Haller, Birbaumer, & Veit, 2010) \| Paired t-test \| Condition difference \| 6 \|  \| 0.07 \| 0.17 \| 0.36 \|  \| 1.19 \| 1.58 \| \| **Mean** \|  \|  \| **29.9** \|  \| **0.24** \| **0.61** \| **0.76** \|  \| **0.77** \| **>0.99** \| \| **Median** \|  \|  \| **22.5** \|  \| **0.15** \| **0.67** \| **0.98** \|  \| **0.58** \| **0.73** \| \| **Mean (all)** \|  \|  \| **22.9** \|  \| **0.22** \| **0.61** \| **0.77** \|  \| **0.76** \| **0.99** \| \| **Median (all)** \|  \|  \| **18.0** \|  \| **0.16** \| **0.70** \| **0.98** \|  \| **0.55** \| **0.71** \| |
| --- | --- | --- | --- | --- | --- | --- | --- | --- | --- | --- | --- | --- | --- | --- | --- | --- | --- | --- | --- | --- | --- | --- | --- | --- | --- | --- | --- | --- | --- | --- | --- | --- | --- | --- | --- | --- | --- | --- | --- | --- | --- | --- | --- | --- | --- | --- | --- | --- | --- | --- | --- | --- | --- | --- | --- | --- | --- | --- | --- | --- | --- | --- | --- | --- | --- | --- | --- | --- | --- | --- | --- | --- | --- | --- | --- | --- | --- | --- | --- | --- | --- | --- | --- | --- | --- | --- | --- | --- | --- | --- | --- | --- | --- | --- | --- | --- | --- | --- | --- | --- | --- | --- | --- | --- | --- | --- | --- | --- | --- | --- | --- | --- | --- | --- | --- | --- | --- | --- | --- | --- | --- | --- | --- | --- | --- | --- | --- | --- | --- | --- | --- | --- | --- | --- | --- | --- | --- | --- | --- | --- | --- | --- | --- | --- | --- | --- | --- | --- | --- | --- | --- | --- | --- | --- | --- | --- | --- | --- | --- | --- | --- | --- | --- | --- | --- | --- | --- | --- | --- | --- | --- | --- | --- | --- | --- | --- | --- | --- | --- | --- | --- | --- | --- | --- | --- | --- | --- | --- | --- | --- | --- | --- | --- | --- | --- | --- | --- | --- | --- | --- | --- | --- | --- | --- | --- | --- | --- | --- | --- | --- | --- | --- | --- | --- | --- | --- | --- | --- | --- | --- | --- | --- | --- | --- | --- | --- | --- | --- | --- | --- | --- | --- | --- | --- | --- | --- | --- | --- | --- | --- | --- | --- | --- | --- | --- | --- | --- | --- | --- | --- | --- | --- | --- | --- | --- | --- | --- | --- | --- | --- | --- | --- | --- | --- | --- | --- | --- | --- | --- | --- | --- | --- | --- | --- | --- | --- | --- | --- | --- | --- | --- | --- | --- | --- | --- | --- | --- | --- | --- | --- | --- | --- | --- | --- | --- | --- | --- | --- | --- | --- | --- | --- | --- | --- | --- | --- | --- | --- | --- | --- | --- | --- | --- | --- | --- | --- | --- | --- | --- | --- | --- | --- | --- | --- | --- | --- | --- | --- | --- | --- | --- | --- | --- | --- | --- | --- | --- | --- | --- | --- | --- | --- | --- | --- | --- | --- | --- | --- | --- | --- | --- | --- | --- | --- | --- | --- | --- | --- | --- | --- | --- | --- | --- | --- | --- | --- | --- | --- | --- | --- | --- | --- | --- | --- | --- | --- | --- | --- | --- | --- | --- | --- | --- | --- | --- | --- | --- | --- | --- | --- | --- | --- | --- | --- | --- | --- | --- | --- | --- | --- | --- | --- | --- | --- | --- | --- | --- | --- | --- | --- | --- | --- | --- | --- | --- | --- | --- | --- | --- | --- | --- | --- | --- | --- | --- | --- | --- | --- | --- | --- | --- | --- | --- | --- | --- | --- | --- | --- | --- | --- | --- | --- | --- | --- | --- | --- | --- | --- | --- | --- | --- | --- | --- | --- | --- | --- | --- | --- | --- | --- | --- | --- | --- | --- | --- | --- | --- | --- | --- | --- | --- | --- | --- | --- | --- | --- | --- | --- | --- | --- | --- | --- | --- | --- | --- | --- | --- | --- | --- | --- | --- | --- | --- | --- | --- | --- | --- | --- | --- | --- | --- | --- | --- | --- | --- | --- | --- | --- | --- | --- | --- | --- | --- | --- | --- | --- | --- | --- | --- | --- | --- | --- | --- | --- | --- | --- | --- | --- | --- | --- | --- | --- | --- | --- | --- | --- | --- | --- | --- | --- | --- | --- | --- | --- | --- | --- | --- | --- | --- | --- | --- | --- | --- | --- | --- | --- | --- | --- | --- | --- | --- | --- | --- | --- | --- | --- | --- | --- | --- | --- | --- | --- | --- | --- | --- | --- | --- | --- | --- | --- | --- | --- | --- | --- | --- | --- | --- | --- | --- | --- | --- | --- | --- | --- | --- | --- | --- | --- | --- | --- | --- | --- | --- | --- | --- | --- | --- | --- | --- | --- | --- | --- | --- | --- | --- | --- | --- | --- | --- | --- | --- | --- | --- | --- | --- | --- | --- |

Note. * = Test inferred; ^#^ = no group comparison. All tests were performed two-tailed, unless otherwise specified. The estimations are not based on actual outcome measures, but rather on sample sizes, liberal assumptions (no multiple comparison correction, high correlations between repeated measures (0.8)) and (mostly) simplified statistical tests.

**Table S2**

***Statistical power and sensitivity for clinical measures of individual studies.***

*Power is estimated (in percentage) for small, medium, and large effects (based on Cohen’s d of 0.2, 0.5, and 0.8, respectively). Sensitivity, or estimation of detected effect size (based on Cohen’s d) with a certain power, is calculated for the power of 80 and 95%. Power and sensitivity calculations for clinical measures include only studies performing a group analysis of clinical improvement. Only studies that performed a group analysis and reported sufficient information to perform a power analysis are included in the table. Two calculations are presented at the bottom of the table: the results in black exclude pilot, proof-of-principle, and feasibility studies (N=11); the results in grey include all studies that performed a group analysis (N=27). Pilot, feasibility, and proof-of-principle studies are marked in grey.*

|  |  |  |  |  | **Power** | | |  | **Sensitivity (in Cohen’s d)** | |
| --- | --- | --- | --- | --- | --- | --- | --- | --- | --- | --- |
| **Publication** | **Test** | **Criterion** | **N** |  | **d = 0.2** | **d = 0.5** | **d = 0.8** |  | **Power = 80%** | **Power = 95%** |
| (Karch et al., 2015) | 2x2 mixed ANOVA | Group (patients true rtfMRI & controls true rtfMRI) X time (before NF & after NF) | 27 |  | 0.35 | 0.98 | 1.00 |  | 0.35 | 0.46 |
| (Karch et al., 2019) | 2x2 mixed ANOVA | Group X Time (pre-post) | 22 |  | 0.29 | 0.94 | 1.00 |  | 0.40 | 0.51 |
| (Hartwell et al., 2016) | 2x3 mixed ANOVA | Group X Time (3 visits) | 44 |  | 0.61 | 1.00 | 1.00 |  | 0.25 | 0.31 |
| (Zilverstand et al., 2017) | 2x2 mixed ANOVA | Group X Time | 13 |  | 0.18 | 0.74 | 0.99 |  | 0.54 | 0.70 |
| (Alegria et al., 2017; Rubia et al., 2019) | 2x2 mixed ANOVA | Group X Time | 31 |  | 0.40 | 0.99 | 1.00 |  | 0.33 | 0.42 |
| (Zilverstand et al., 2015) | 2x4 mixed ANOVA | Group X Time (screening, post- fMRI, 2-week, 3-month) | 18 |  | 0.30 | 0.98 | 1.00 |  | 0.36 | 0.46 |
| (Paret et al., 2016) | RM ANOVA | 4 RM | 10 |  | 0.17 | 0.80 | 1.00 |  | 0.50 | 0.63 |
| (Zaehringer et al., 2019) | RM ANOVA | 3 RM | 25 |  | 0.37 | 0.99 | 1.00 |  | 0.33 | 0.42 |
| (Robineau et al., 2019) | RM ANOVA^#^ | 3 RM | 6 |  | 0.10 | 0.43 | 0.83 |  | 0.77 | 0.97 |
| (Yuan et al., 2014) | Independent t-test* | Group difference | 27 |  | 0.08 | 0.24 | 0.51 |  | 1.12 | 1.45 |
| (Young et al., 2014) | Independent t-test (1-tailed)* | Group difference | 21 |  | 0.11 | 0.27 | 0.51 |  | 1.19 | 1.58 |
| (Young, Misaki, et al., 2017; Young et al., 2018; Young, Siegle, et al., 2017) | 2x4 mixed ANOVA | Group X Visit (4) | 36 |  | 0.58 | 1.00 | 1.00 |  | 0.25 | 0.31 |
| (Zotev et al., 2016) | Independent t-test* | Group difference | 24 |  | 0.08 | 0.21 | 0.46 |  | 1.20 | 1.55 |
| (Zotev et al., 2019) | Independent t-test* | Group difference | 24 |  | 0.07 | 0.20 | 0.42 |  | 1.27 | 1.63 |
| (Linden et al., 2012) | 2x2 mixed ANOVA | Group X Time (pre-post) | 16 |  | 0.22 | 0.84 | 1.00 |  | 0.48 | 0.61 |
| (Mehler et al., 2018) | 2x2 mixed ANOVA | Group X Time (pre-post) | 32 |  | 0.41 | 0.99 | 1.00 |  | 0.32 | 0.42 |
| (Jaeckle et al., 2019) | 2x2 mixed ANOVA | Group X Time (pre-post) | 40 |  | 0.50 | 1.00 | 1.00 |  | 0.29 | 0.37 |
| (Zahn et al., 2019) | 2x2 mixed ANOVA | Group X Time | 28 |  | 0.36 | 0.98 | 1.00 |  | 0.35 | 0.45 |
| (Rance et al., 2018) | 2x2 mixed ANOVA | Group X Time (pre-post) | 37 |  | 0.46 | 1.00 | 1.00 |  | 0.30 | 0.39 |
| (Hohenfeld et al., 2017) | Paired t-test* | Pre-post | 10 |  | 0.09 | 0.29 | 0.62 |  | 1.00 | 1.29 |
| (Tinaz et al., 2018) | Paired t-test^#^ | Pre-post | 8 |  | 0.08 | 0.23 | 0.50 |  | 1.16 | 1.50 |
| (Subramanian et al., 2011) | Paired t-test (one-tailed) ^#^ | Pre-post | 5 |  | 0.10 | 0.24 | 0.44 |  | 1.36 | 1.82 |
| (Subramanian et al., 2016) | ANCOVA | Group difference | 30 |  | 0.08 | 0.26 | 0.56 |  | 1.06 | 1.37 |
| (Zweerings et al., 2018) | Paired t-test | Group difference | 8 |  | 0.08 | 0.23 | 0.50 |  | 1.16 | 1.50 |
| (Zotev et al., 2018b) | Paired t-test^#^ | Pre-post | 15 |  | 0.11 | 0.44 | 0.82 |  | 0.78 | 1.00 |
| (Orlov et al., 2018) | Paired t-test | Pre-post | 11 |  | 0.09 | 0.32 | 0.67 |  | 0.94 | 1.21 |
| (Emmert et al., 2017) | 2x3 mixed ANOVA | Group X Time (pre, post, follow up) | 14 |  | 0.21 | 0.87 | 1.00 |  | 0.46 | 0.58 |
| **Mean** |  |  | **26.7** |  | **0.31** | **0.73** | **0.85** |  | **0.58** | **0.74** |
| **Median** |  |  | **27.0** |  | **0.30** | **0.98** | **>0.99** |  | **0.36** | **0.46** |
| **Mean (all)** |  |  | **21.6** |  | **0.24** | **0.65** | **0.81** |  | **0.69** | **0.88** |
| **Median (all)** |  |  | **22.0** |  | **0.18** | **0.80** | **1.00** |  | **0.50** | **0.63** |

Note. * = test inferred; ^#^ = no group comparison. All tests were performed two-tailed, unless otherwise specified. The estimations are not based on actual outcome measures, but rather on sample sizes, liberal assumptions (no multiple comparison correction, high correlations between repeated measures (0.8)) and (mostly) simplified statistical tests.

**Table 2**

***Details of all 62 clinical fMRI neurofeedback studies found in an extensive search.***

*Studies are sorted based on the clinical population and are categorized based on their methods and results. Next to each category, a corresponding item number from CRED-NF (see table 1) is noted in brackets.*

| Publication (1b) | Clinical population | Clinical group size^3^ | ROI | Strategy (3b) | Transfer run(s) and success | Control  /comparison^11^ group (2a, 2b) | Regulation direction and success (5a) | Experimental vs control regulation (5c) | Regulation plots per run/session (5b) | Symptom measures (6a) | Regulation and clinical correlation (6b) | Follow-up | Registered trial (1a) |
| --- | --- | --- | --- | --- | --- | --- | --- | --- | --- | --- | --- | --- | --- |
| (Canterberry et al., 2013) | Addiction | 9 | ACC | No | No | - | 🡫 Yes | - | Sess | Beh ~ | No | - | - |
| (Hanlon et al., 2013) | Addiction | 15 | vACC and dmPFC | No | No | - | 🡫 Yes | - | - | Beh - | - | - | - |
| (Li et al., 2013) $ | Addiction | 12 | ACC and mPFC | No | No | - | 🡫🡩_diff_ Yes (ACC) | - | - | Beh 🡩 | Yes | - | - |
| (Karch et al., 2015) $ | Addiction | 13 | ACC, dlPFC, or insula | No | No | Multi. Healthy NFB & SHAM, Clin SHAM 🗶 | 🡫 Yes | No, two groups | - | Clin 🗶 | - | - | - |
| (Karch et al., 2019) | Addiction | 22^4^ | ACC, dlPFC, or insula | No | No | Clin Diff ROI - | n.a. ^13^ | n.a. | - | Clin 🗶 | - | - | - |
| (Kim et al., 2015) | Addiction | 7 | # Bilateral ACC, medial pFC and OFC | No | Yes ✓ | Clin Diff con ✓ | 🡩Yes | No (con>exp) | Sess | Beh 🗶 | - | - | - |
| (Hartwell et al., 2016) | Addiction | 21 | PFC | Sug | No | Clin No NFB 🗶 | 🡫 Yes | Yes | Sess | Clin ~ | - | - | - |
| (Kirschner et al., 2018) | Addiction | 22 | # VTA and SN | Yes | Yes 🗶 | Healthy NFB ✓ | 🡩Yes | No | Yes | - | - | - | - |
| (Zilverstand et al., 2017) $ | ADHD | 7 | dACC | Yes | Yes ✓ | * Clin Unaware ✓ | 🡩Yes | No | Sess | Clin 🡩 | - | - | ISRCTN12390961 |
| (Alegria et al., 2017; Rubia et al., 2019) $ | ADHD | 18 | # rIFG | No | Yes ✓ | * Clin Diff ROI ✓ | 🡩Yes | Yes | Pre-post | Clin 🡩 | Yes | >5m – clin 🡩 | ISRCTN12800253 |
| (Buyukturkoglu et al., 2015) $ | Anxiety | 3 | Bilateral AI | No | Yes ✓ | - | 🡫 Yes | - | Yes | Clin 🡩 | - | - | - |
| (Zilverstand et al., 2015) | Anxiety | 9 | Insula and dlPFC | Yes | No | * Clin Unaware ✓ | 🡫🡩_diff_ Yes | Yes | Yes | Clin 🡩 | Yes | 3m – clin 🡩 | - |
| (Scheinost et al., 2013) | Anxiety | 12 | OFC | Yes | Yes ✓ | Clin Yoked 🗶 | 🡫🡩_same_ Yes | No | Pre-post | Beh 🡩 | Yes | - | - |
| (Scheinost et al., 2014) $ | Anxiety | 5 | OFC | Yes | Yes - | Healthy NFB - | 🡫🡩_same_ - | - | - | Clin 🡩 | - | - | - |
| (Sreedharan et al., 2019b, 2019a) | Aphasia | 4 | Broca and Wernicke's areas | Sug | No | Multi. Healthy NFB and Clin No training ✓ | 🡩Yes | - | Sess | Beh 🗶 | - | - | - |
| (Ramot et al., 2017) $ | Autistic disorder | 17 | STS, SSC and IPL^8^ | No | No | * Clin Diff con 🗶 | 🡩Yes | Yes | Sess | Clin 🗶 | - | 5-56w - conn 🡩 | NCT01031407 |
| (Paret et al., 2016) $ | BPD | 10 | Bilateral amygdala | No | Yes 🗶 | - | 🡫 Yes | - | Sess | Clin 🗶 | - | - | - |
| (Zaehringer et al., 2019) ^ | BPD | 25 | # Right amygdala | No | No | - | 🡫 Yes | - | Sess | Clin 🡩 | No | 6w - clin 🡩 | NCT02866110 DRKS00009363 |
| (Liew et al., 2016) $ | Brain damage | 4 | M1 and ipsilateral thalamus | Sug | Yes ✓^9^ | - | 🡩Yes (3/4) | - | Pre-post | - | - | - | - |
| (Sitaram et al., 2012) $ | Brain damage | 2 | PMv | No | Yes ✓ | Healthy NFB ✓ | 🡩Yes | - | Sess | Beh ^18^ 🗶 | - | - | - |
| (Robineau et al., 2019) $ | Brain damage | 6 | V1 | Sug | No | Clin Diff con 🗶 | 🡩Yes | - | Sess | Clin 🡩 | No | - | - |
| Publication (1b) | *Clinical population* | *Clinical group size^3^* | *ROI* | *Strategy (3b)* | *Transfer run(s) and success* | *Control*  */comparison^11^ group (2a, 2b)* | *Regulation direction and success (5a)* | *Experimental vs control regulation (5c)* | *Regulation plots per run/session (5b)* | *Symptom measures (6a)* | *Regulation and clinical correlation (6b)* | *Follow-up* | *Registered trial (1a)* |
| (DeCharms et al., 2005) | Chronic pain | 8 | rACC | Sug | No | Multi. Clin NFB and healthy No NFB, No NFB diff str, NFB diff ROI, yoked ✓ | 🡫🡩_same_ Yes | - | Yes | Beh 🡩 | Yes | - | - |
| (Guan et al., 2015) | Chronic pain | 8 | rACC | Sug | No | ** Clin Diff ROI 🗶 | 🡫🡩_same_ Yes | Yes | - | Beh 🡩 | No | - | - |
| (MacDuffie et al., 2018) $ | Depression | 13 | ACC | Yes | No | Clin WS - | 🡫 n.a.^14^ | n.a. | n.a. | - | - | - | - |
| (Hamilton et al., 2016) $ | Depression | 12 | Fronto-insular cortex and dACC | Yes | Yes ✓ | * Clin Yoked ✓ | 🡫 Yes | No | Pre-post | - | - | - | - |
| (Yuan et al., 2014) | Depression | 14 | # Left amygdala | Yes | Yes - | ** Multi. Healthy NFB and Clin Diff ROI - | 🡩- | - | - | Clin 🡩 | - | - | - |
| (Young et al., 2014) $ | Depression | 14 | # Left amygdala | Yes | Yes ✓ | ** Clin Diff ROI 🗶 | 🡩Yes | Yes | Yes | Clin 🡩 | - | - | - |
| (Young et al., 2018, 2017b, 2017a) | Depression | 19 | # Left amygdala | Yes | Yes ✓ | ** Clin Diff ROI ✓ | 🡩Yes | Yes | Yes | Clin 🡩 | Yes | - | NCT02079610 |
| (Zotev et al., 2016) $ | Depression | 13 | # Left amygdala | Yes | Yes ✓ | ** Clin Diff ROI 🗶 | 🡩Yes | Yes | Yes | Clin 🡩 | - | - | - |
| (Zotev et al., 2019) ^$ | Depression | 16 | # Left amygdala and left rACC | Yes | Yes ✓ | * Clin SHAM-C NFB 🗶 | 🡩Yes | Yes | Yes | Clin 🡩 | Yes | - | - |
| (Linden et al., 2012) $ | Depression | 8 | Various regions: dlPFC, vlPFC, insula | No | No | Clin MR - | 🡩Yes | - | Yes | Clin 🡩 | Yes | - | - |
| (Peciña et al., 2018) | Depression^1^ | 24 | - | - | No | * - | n.a. | - | n.a. | - | - | - | - |
| (Mehler et al., 2018)@ | Depression | 16 | Mainly anterior brain areas such as insula and striatum | Sug | Yes 🗶 | * Clin Diff str ✓ | 🡩Yes | No | Sess | Clin 🡩 | - | 18w - clin 🡩 | NCT01544205 |
| (Jaeckle et al., 2019) ^@$ | Depression | 19 | rSATL and pSCC | No | No | * Clin Diff str ✓ | 🡫 Yes | - | Yes | Clin 🡩 | No | - | ISRCTN10526888 |
| (Zahn et al., 2019) $ | Depression^2^ | 14 | aSCC and aSTC | No | No | ** Clin Diff con 🗶 | 🡩Yes | Yes | Pre-post | Clin - | - | - | NCT01920490 |
| (Rance et al., 2018) | Misc | 10 (A),  20 (TS) ^5^ | OFC (A), SMA (TS) | Yes | - | ** Clin Yoked - | 🡫🡩_same_ - | - | - | Clin 🡩 | - | 2, 4, 6, 8w - clin 🡩 | NCT02206945 NCT01702077 |
| (McDonald et al., 2017) | Misc | 76 ^6^ | DMN | Yes | No | - | 🡫🡩_same_ Yes | - | - (n.a.) | - | - | - | - |
| (Skouras and Scharnowski, 2019)@ | Misc | 74 | DMN | Yes | No | Healthy NFB ✓ | 🡫🡩_same_ Yes | Yes | Yes | - | - | - | - |
| (Hohenfeld et al., 2017) $ | ND-A | 10 | PHG | Yes | No | Multi. Healthy NFB & SHAM ✓ | 🡩Yes | No | Yes | Clin 🡩 | - | - | - |
| (Papoutsi et al., 2018b) $ | ND-H | 10 | SMA | Sug | No | - | 🡩Yes | - | Sess, pre-post | Beh 🗶 | - | - | - |
| (Papoutsi et al., 2018a) ^$ | ND-H | 8 & 8 ^7^ | SMA | Sug | Yes ✓ | * Multi. Clin 2x SHAM (activity ✓ and connectivity 🗶) | 🡩Yes ^15^ | No | Yes | Beh 🗶 | - | 3x: 2, 4-6 and 8-10w (beh & trans 🗶) | - |
| Publication (1b) | *Clinical population* | *Clinical group size^3^* | *ROI* | *Strategy (3b)* | *Transfer run(s) and success* | *Control*  */comparison^11^ group (2a, 2b)* | *Regulation direction and success (5a)* | *Experimental vs control regulation (5c)* | *Regulation plots per run/session (5b)* | *Symptom measures (6a)* | *Regulation and clinical correlation (6b)* | *Follow-up* | *Registered trial (1a)* |
| (Tinaz et al., 2018) $ | ND-P | 8 | Right insula and dlPFC | Yes | Yes ✓ | Clin No NFB - | 🡩Yes | - | Yes | Clin 🗶 | - | - | - |
| (Subramanian et al., 2011) $ | ND-P | 5 | SMA | Sug | No | Clin No NFB ✓ | 🡩Yes | - | Yes | Clin 🡩 | - | 2w (beh 🡩) | - |
| (Buyukturkoglu et al., 2013) $ | ND-P | 1 | SMA | Sug | No | Healthy NFB ✓ | 🡩Yes | - | - | Beh 🗶 | - | - | - |
| (Subramanian et al., 2016) | ND-P | 15 | SMA | Yes | Yes ✓ | Clin No NFB - | 🡩Yes | - | Sess | Clin 🡩 | No | - | NCT01867827 |
| (Frank et al., 2012) | Obesity | 10 | # Bilateral AI | Sug | No | Healthy NFB ✓ | 🡩Yes | Yes | - | - | - | - | - |
| (Spetter et al., 2017) $ | Obesity | 8 | dlPFC and vmPFC | Sug | No | - | 🡩Yes | - | Sess | Beh 🗶 | - | - | - |
| (Kohl et al., 2019b) | Obesity | 17 | Left dlPFC | No | No | * Clin Diff ROI ✓^12^ | 🡩Yes | No | Yes | Beh 🡩 | Yes | 4w (beh 🡩) | NCT02148770 |
| (Sitaram et al., 2014) $ | PP | 4 | Left AI | Sug | No | - | 🡩No ^16^ | - | Yes | Beh ~ | - | - | - |
| (Zweerings et al., 2018) | PTSD | 9 | # ACC | Sug | Yes ✓ | Healthy NFB ✓ | 🡩Yes | No (cont>exp) | - | Clin 🡩 | - | - | - |
| (Gerin et al., 2016) $ | PTSD | 3 | Amygdala | No | No | - | 🡫 Yes | - | - | Clin 🡩 | - | - | - |
| (Nicholson et al., 2017) | PTSD | 10 | Bilateral amygdala | No | Yes ✓ | - | 🡫 Yes | - | Yes | - | - | - | - |
| (Nicholson et al., 2018) | PTSD | 14 | Bilateral amygdala | No | Yes ✓ | - | 🡫 Yes | - | Yes | - | - | - | - |
| (Misaki et al., 2018) | PTSD | 16 | # Left amygdala | Sug | Yes - | * Multi. Clin Diff ROI and healthy veterans NFB ✓ | 🡩Yes | - | Yes | Clin 🡩 | - | - | - |
| (Zotev et al., 2018b) | PTSD | 15 | # Left amygdala | Sug | Yes 🗶 | * Clin Diff ROI 🗶 | 🡩Yes | No | Yes | Clin 🡩 | Yes | - | - |
| (Cordes et al., 2015) | Schizophrenia | 11 | # ACC | Sug | No | Healthy NFB ✓ | 🡩Yes | Yes | - | - | - | - | - |
| (Dyck et al., 2016) $ | Schizophrenia | 3 | # ACC | Sug | Yes ✓^10^ | - | 🡩Yes | - | Sess | Clin ~ | - | - | - |
| (Zweerings et al., 2019) | Schizophrenia | 21 | Left IFG and left pSTG | No | No | ** Healthy NFB ✓ | 🡫🡩_same_ Yes | No | - | Beh - | - | - | - |
| (Ruiz et al., 2013) | Schizophrenia | 9 | Bilateral AI | Sug | Yes 🗶 | - | 🡩Yes | - | Yes | Beh 🡩 | Yes | - | - |
| (Orlov et al., 2018) $ | Schizophrenia | 12 | Left STG | No | Yes ✓ | - | 🡫 Yes | - | Yes | Clin 🗶 | - | - | - |
| (Emmert et al., 2017) $ | Tinnitus | 7 | AC | Sug | Yes - | Clin NFB (inter 🗶) | 🡫 Yes ^17^ | No | Sess | Clin ~ | - | 6w (clin 🗶) | - |
| (Haller et al., 2010) $ | Tinnitus | 6 | AC | No | No | - | 🡫 Yes (5/6) | - | Yes | Beh ~ | - | - | - |

Legend per category:

*Publication:* ^ = not peer-reviewed at the time of the search; @ = sample size calculation, $ = pilot, feasibility, or proof-of-principle

*Clinical population:* ADHD = attention deficit hyperactivity disorder; Aphasia = Expressive (Broca’s) aphasia; BPD = borderline personality disorder; PP = psychopathy; Misc = miscellaneous; ND-A = neurodegenerative disease (Alzheimer’s disease); ND-H = neurodegenerative disease (Huntington’s disease); ND-P = neurodegenerative disease (Parkinson’s disease); PTSD = post-traumatic stress disorder; ^1^ Placebo study; ^2^ The patients were remitted

*Clinical Group Size:* ^3^The size of the experimental group (one arm only). ^4^ The article presents only the data from the experimental group (n=22) of a previous neurofeedback study, which they split into relapsed (12) and non-relapsed group (10); ^5^ A = anxiety, TS = Tourette’s syndrome; ^6^ They performed analysis also on 121 participants, but here we report only the group of the participants that did not fall asleep; ^7^ One group received activity- and one group connectivity-based neurofeedback

*ROI:* # anatomical localizer; ^8^ Based on a previous study

*Strategy:* Sug = suggestions

*Transfer run(s) and success:* tick (✓) for yes, cross (🗶) for no, minus (-) for not stated; ^9^ in 2/4 participants; ^10^ in 1/3 participants

*Control/comparison group:* ^11^ Comparison group refers to healthy participants, who do not control for the effects of neurofeedback (for further explanation see the results section (item 2a)); * = single-blinded, ** = double-blinded; regulation success: tick (✓) for yes, cross (🗶) for no, minus (-) for not stated; ^12^ Behavioral testing was also blinded; Clin = the same clinical population; NFB = neurofeedback; Multi = more than one control group; Diff ROI = different region of interest; diff str = different strategy; diff con = different connectivity; RM = mental rehearsal; SHAM-C = computer generated SHAM

*Regulation Success*: 🡫 = down-regulation; 🡩 = up-regulation; 🡫🡩_diff_ = bidirectional regulation of a different region; 🡫🡩_same_ = bidirectional regulation of the same region; ^13^ reported in an unpublished paper; ^14^ The participants could regulate, but the regulation was not the main interest of the study; ^15^ Yes in the activity group and no in the connectivity group; ^16^ one participant could successfully regulate, but with more sessions; ^17^ The group receiving continuous feedback could regulate, the control group receiving intermittent feedback could not

*Experimental vs Control:* cont>exp = control group significantly better regulated than the experimental group

*Plots:* Sess = average session regulation; Pre-post = regulation before and after training; Yes = runs and session were represented; - = no plots

*Symptom measures:* Clin = clinical measure; Beh = behavioral measure; - = not reported; 🡩 = improvement; 🗶 = no improvement; ~ = mixed results (some participants improved or there was an improvement over each session, but not across sessions); ^18^ Measured with TMS

*Correlation:* Correlation between symptom improvement and regulation success

*Follow-up:* duration between the last session and follow-up session expressed in weeks (w) or months (m); clin = clinical measure; beh = behavioral measure; trans = transfer run; conn = connectivity changes; 🡩 = stable improvement; 🗶 = diminished improvement

*Registered:* the registration identifier

**References**

Alegria, A.A., Wulff, M., Brinson, H., Barker, G.J., Norman, L.J., Brandeis, D., Stahl, D., David, A.S., Taylor, E., Giampietro, V., Rubia, K., 2017. Real-time fMRI neurofeedback in adolescents with attention deficit hyperactivity disorder. Hum. Brain Mapp. 38, 3190–3209. https://doi.org/10.1002/hbm.23584

Calamia, M., Markon, K., Tranel, D., 2013. The robust reliability of neuropsychological measures: Meta-analyses of test-retest correlations. Clin. Neuropsychol. 27, 1077–1105. https://doi.org/10.1080/13854046.2013.809795

Cohen, J., 1992. A Power Primer. Psychol. Bull. 112, 155–159. https://doi.org/10.1037/0033-2909.112.1.155

Cohen, J., 1988. Statistical Power Analysis for the Behavioral Sciences, 2nd editio. ed. Lawrence Erlbaum Associates, Inc., Hillsdale, NJ.

Rubia, K., Criaud, M., Wulff, M., Alegria, A., Brinson, H., Barker, G., Stahl, D., Giampietro, V., 2019. Functional connectivity changes associated with fMRI neurofeedback of right inferior frontal cortex in adolescents with ADHD. Neuroimage 188, 43–58. https://doi.org/10.1016/j.neuroimage.2018.11.055
